# Enhancement of infrared absorption through a patterned thin film of magnetic field and spin-coating directed self-assembly of gold nanoparticle stabilised ferrofluid emulsion†

**DOI:** 10.1039/d3ra01369c

**Published:** 2023-08-10

**Authors:** Paul Okpozo, Yashashchandra Dwivedi, Dehong Huo, Ketan Pancholi

**Affiliations:** a School of Engineering, Sir Ian Wood Building, Robert Gordon University Garthdee Aberdeen AB10 7GJ UK k.pancholi2@rgu.ac.uk; b Physics Department, National Institute of Technology Kurukshetra Kurukshetra 136119 India; c School of Engineering, Newcastle University Newcastle NE1 7RU UK; d Advanced Materials Group, School of Engineering, Robert Gordon University Aberdeen UK

## Abstract

Molecular vibration signals were amplified by the gold strip gratings as a result of grating resonances and nearby electric field hotspots. Colloidal gold island films exhibit similar enhancement; however, the uneven geometrical characteristics of these films restrict the tunability of the vibrational enhancement. Infrared absorption is enhanced by regular metallic patterns such as arrays of strips fabricated using a top-down approach such as nanolithography, although this technology is expensive and difficult. The significant infrared absorption may serve as tuneable antenna sensitization to improve the sensor performance. In this article, we present a simple one-step process for fabricating optically sensitive ordered arrays of a gold nanoparticle ferrofluid emulsion in polyvinyl alcohol (PVA) using a magnetic field-directed and spin-coating self-assembly (MDSCSA) process. Techniques such as UV-visible absorption, scanning electron microscopy, and grazing-angle infrared spectroscopy were used to evaluate various parameters associated with the nanostructures. Unlike the gold strips, the chain-like features in the iron oxide nanoparticle arrays were discontinuous. The fabricated chain-like ordered arrays have been shown to increase the local field to enhance the infrared absorption corresponding to the symmetric vibration of the –CH_2_ (2918 cm^−1^) group present in PVA by ∼667% at a 45° grazing angle, as the chain thickness (CT) increased by 178%. This scalable and simple method can potentially generate low-cost patterns for antenna sensitisation.

## Abstract 2

Molecular vibration signals were amplified by the gold strip gratings as a result of grating resonances and nearby electric field hotspots.

## Graphical abstract

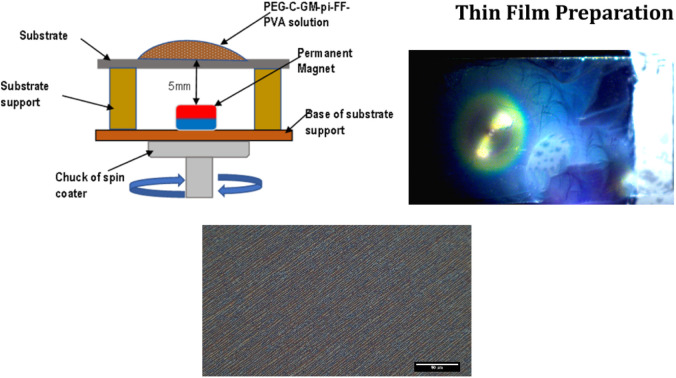

## 1 Introduction

Infrared (IR) spectroscopy is a rapid, accurate, and robust method for the detection of molecules in a range of applications. For instance, its integration with microfluidics has enabled the detection of vascular endothelial growth factor (VEGF) for early disease diagnosis.^1,2^ However, the sensitivity of IR spectroscopy is limited when detecting trace amounts of analytes, owing to the small cross-section of its molecular vibrational signals. Surface-enhanced infrared absorption spectroscopy (SEIRA) is commonly used to improve sensitivity. Metal nanostructures, such as gold gratings, have been found to be particularly useful in SEIRA, as the plasmon polariton resonance of gold gratings in the mid-IR region can be matched with molecular vibrational signals to enhance sensitivity. However, fabricating metal nanostructures with precise geometrical features requires time-consuming, complex, and expensive methods such as gold sputtering, electron beam lithography, and nanoimprint lithography. The cost, complexity and scalability may improve by using a simple, rapid, and less complicated magnetically directed self-assembly (MDSA) technique.^3,4^ Although MDSA can non-intrusively organize magnetic nanoparticles (NPs) to form arrays of chains^5,6^ suitable for applications in several fields such as photonics,^7–9^ storage devices,^10^ microfluidics,^11*a*,*b*^ and optical filters,^12^ it is difficult to organize poorly magnetic-responsive (diamagnetic) materials such as gold NPs. Also, the loss of materials from spin coating is a common demerit regarding this fabrication technique.^13^ Combining non-magnetic and optically active materials with magnetic iron oxide helps overcome the limitations of MDSA and enables the formation of arrays of non-magnetic colloidal assemblies in the form of grating order that is suitable for optical applications and performance in terms of diffraction and wave dispersion.^14–16^ This makes this technique advantageous to other homogeneous metal-polymer-composite thin film layer, however, the irregular and jagged NP chains formed during MDSA processing, coupled with the optically inactive iron oxide NPs, may not produce an optical response similar to that of the nanoimprinted gold strips. Additionally, controlling the film thickness produced using MDSA is not ideal. To address these challenges, in this work, the MDSA technique was modified to combine with spin-coating to form an array of gold nanoparticle stabilised pickering ferrofluid emulsion chains to study the feasibility of low-cost antenna for surface enhanced infrared absorption (SEIRA) spectroscopy. The modified MDSA technique, now termed magnetic directed and spin coating self-assembly (MDSCSA), helped to achieve long-range assemblies of gold NPs in the form of arrays of chains as the non-magnetic gold NPs adsorbed at the interfaces^17*b*,*c*^ of the oil ferrofluid oil-in-polyvinyl alcohol aqueous solution nano-emulsion droplets, also known as pickering emulsions aligned to external magnetic field.^18^

It is hypothesized that the clusters of gold and iron oxide NPs would contribute to the electron dynamics to enhance the local electric field, leading to an increase in the infrared-induced molecular vibrational signal^19–21^ and contribute to random light scattering^22^ or interference.^23,24^ The interfacial interaction between gold and iron oxide causes the diffusion of the excited electrons from the Fermi level of gold NPs to the conduction band of the iron oxide, causing charge accumulation at the defect sites of the interface, therefore improving the optical activity of iron oxide.^25,26^ It is expected that the iron oxide in the ferrofluid droplet will interact with the adsorbed gold NPs and localize the electrons to increase the infrared molecular vibrational signal. The advantage of iron oxide as an electron reservoir creates an opportunity for minimizing the required gold concentration in the entire composite system.^26,27^ Therefore, it is beneficial to maintain the required concentration of gold NPs in the chains, the appropriate length and thickness of the chains, and gaps between chains. During magnetic field directed self-assembly, the dipole–dipole interaction between magnetic particles or magnetic fluid droplets builds arrays of chain-like clusters that can span over a long distance at the microscale along the generated flux lines of the magnetic source.^28,29^ The density of these flux lines is dependent on both the size and strength of the magnetic field;^30^ therefore, the chain length, thickness, and gaps between them can be controlled by suitably selecting the magnetic field strength, orientation, and distance from the subject. Additionally, these arrays of chains must be immobilized on the substrate, either by drying or curing an aqueous polymer.^31,32^ The composition considered in this study was an oil-based ferrofluid (hydrophobic phase) dispersed in polyvinyl alcohol (hydrophilic phase). This system tends to maintain the sphericity of the droplets and provides flexibility for the containing iron NPs to align and drive droplets to form chain arrays with respect to the magnetic field direction, as well as to maintain electrostatic, stearic, and viscous hindrance between droplets, preventing them from coalescing.^33,34^ Polyvinyl alcohol (PVA) system can act as an absorber for analyte molecules in such composite systems for sensory applications, like in the case of silver-PVA composite system for the detection of amines.^35^

The applied magnetic field can control the chain arrays and degree of orderliness; however, the introduction of spin coating contributes to the formation of thin-film mono-layered chain arrays. Spin coatings can also contribute to the rapid drying of most hydrophilic polymers.^36–39^ In this process, the contention between the magnetic force pulling the droplets towards the magnetic source and the inertial centrifugal hydrodynamic force of the fluid from the substrate spinning affects the distribution of the droplets and their chain clusters. Therefore, the process was optimized to control the chain morphology. This method allows the formation of a precise nanostructure that controls the plasmon excitation modes and shifts in infrared absorption at different incidence angles of light. This outcome is useful for the development of sensitive biomedical sensors.^40–42^

Comparatively with MDSCSA, other patterned thin film nanofabrication techniques require costly equipment and, in most cases, require clean room to build lithographic template for guiding nanoparticles into arrays.^43,44^ Also, the application of spin coating in MDSCSA is one of the quickest methods for creating thin films, thus making product throughput high.^43,45^ Amongst other established fabrication techniques, nanoimprinting for example, is one of the fastest and most pristine methods for building patterns.^46,47^ However, MDSCSA potentially offers rapid flexibility by switching magnetic configurations and orientations to form different patterns, while nanoimprint would require redesign of template using costly lithographic equipment.^48^

## 2 Methods

This section is divided into two main sub-sections. The first sub-section explains the preparation of gold nanoparticles stabilized pickering ferrofluid emulsion. However, this sub-section involves many steps such as the preparation of oleic coated iron oxide nanoparticles, ferrofluid, ferrofluid emulsion, polyethylene glycol 40S coated gold nanoparticles (PEG-C-GM) through colloidal route and finally, PEG-C-GM nanoparticle stabilized pickering ferrofluid emulsion (PEG-C-GM-pi-FF) in a PVA aqueous solution. In the second sub-section, PEG-C-GM-pi-FF emulsion dispersed in PVA aqueous solution was used to prepare the thin film using MDSCSA method. The thin film contained the array of parallel chains of PEG-C-GM-pi-FF emulsion droplets.

### 2.1 Gold nanoparticle stabilised pickering ferrofluid emulsion preparation

**Fig. 1:**
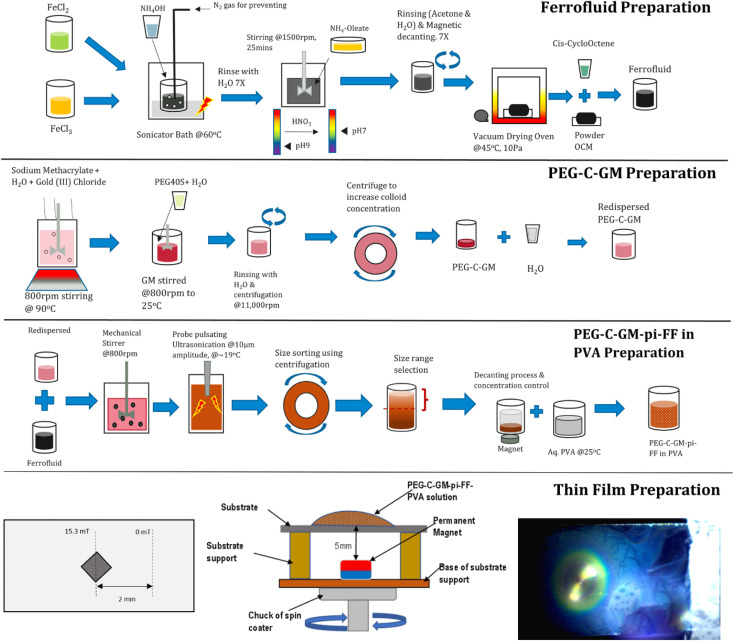
Schematic showing three stages of PEG-C-GM-pi-FF nano-emulsion manufacturing process and thin film preparation.

As seen in Fig. 1, oil-based ferrofluid^49–51^ and gold methacrylate colloid were prepared (ESI S1.18.1 and S.1.18.2†). Subsequently, the PEG-C-GM nanoparticles were separated and dispersed in DI water. All materials and equipment are listed in ESI S1.17 and S.1.17.1.† The prepared aqueous suspension of PEG-C-GM (4 mL) was dispersed in 20 mL of DI water in a sonicator bath for 5 min. Afterwards, 70 μL of oil based ferrofluid was added, and the resulting mixture was stirred at 800 rpm using a rotor-stator for 8 min to create micron-sized emulsion droplets (∼400 μm diameter) of ferrofluid. Furthermore, it was irradiated for 10 minutes with trains of ultrasound pulses with a central frequency of 20 kHz using a probe-type sonicator, MSE® Soniprep 150, to reduce the diameter of the ferrofluid emulsion droplets. Each pulse duration was maintained at 10 seconds, while the transducer displacement was 10 μm. The system was kept cool (∼19 ± 2 °C) by inserting it into a thermo-regulating jacket. Six more batches were prepared using the same procedure. To reduce the degree of polydispersity, 15 millilitres of emulsion were centrifuged at a precisely determined speed of 1000 revolutions per minute for 10 minutes. Prior to determining the optimal speed and time of the size control process, a series of tests were conducted to optimize the size distribution. This nanoemulsion was termed polyethylene glycol-coated gold methacrylate pickering ferrofluid (PEG-C-GM-pi-FF). This process is shown in the third row of Fig. 1.

Separately, the 5 wt%, 3.3 wt%, 1.7 wt% and 1.3 wt% of aqueous PVA solutions were prepared by adding PVA powder into DI water and stirring at 200 rpm for 10 minutes. The densities and viscosities of the resulting solutions with different concentrations are presented in ESI Table S2.† Subsequently, the solutions were heated at approximately 80 °C for 2.5 hours until they turned transparent. Five millilitres of the pickering emulsion were then added to 10 mL of an aqueous solution of PVA and stirred at 400 rpm for 1 hour using a rotor mixer.

### 2.2 Thin film preparation using MDSCSA method

**Fig. 2:**
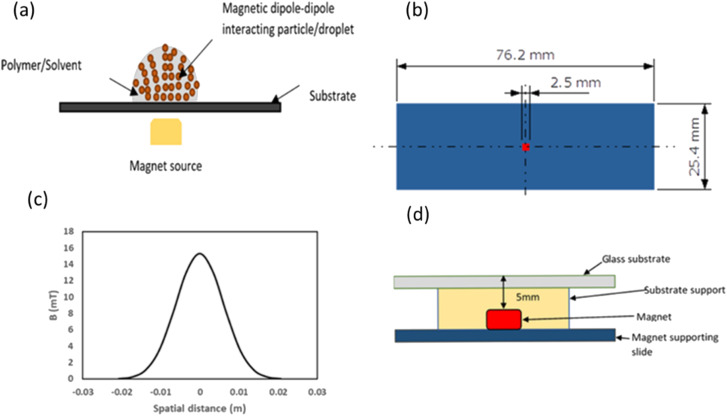
(a) Schematic representation of the magnetized droplet of gold nanoparticle-stabilized pickering ferrofluid emulsion (PEG-C-GM-pi-FF) in an aqueous PVA solution. (b) Top view of the substrate with dimensions. (c) Measured spatial distribution of the magnetic field on the glass substrate surface. (d) Cross-sectional view of the experimental setup used for measuring magnetic fields and preparing magnetic field-directed self-assembly of PEG-C-GM-pi-FF *via* spin coating. The glass slide supporting magnet is represented by the blue feature, and the magnet itself is depicted as a red square. The distance between the magnet and the glass substrate is 5 mm.

As shown in Fig. 2a–c, the experimental setup was designed to prepare the thin film containing arrays of chains of gold nanoparticle-stabilized pickering ferrofluid droplets. To prepare the thin film, a substrate was fixed on the base with the help of a support. The magnet was placed approximately 5 mm below the surface of the substrate as shown in Fig. 2d, and it was at the centre of the substrate as well (a top-view in Fig. 2b). A strong cubic neodymium magnet having 2.5 mm length and magnetic field strength of 202 mT on surface was placed beneath the surface of the substrate measuring 76.2 mm × 25.4 mm (Fig. 2b). Fig. 2c shows the magnetic field distribution in mT across the substrate.

The setup was spun using an SCS™ 6800 spin coater at a range of speeds programmed to operate within a specified timeframe. The spin coater was set to spin at the maximum speed for 50 seconds after accelerating for 5 seconds. Finally, the spinner was decelerated for 5 seconds to bring it to a standstill, making it a total spinning time of 60 seconds. The glass slide substrates were cleaned to remove debris on the surface with 70 wt% aqueous isopropanol solution and then dried in an oven at 60 °C for one hour. The effects of this treatment were tested using contact-angle measurements. Prior to starting the spinning process, 0.5 mL of PEG-C-GM-pi-FF emulsion dispersed in PVA aqueous solution was allowed to settle on the substrate for 60 seconds, providing sufficient time for the droplets to interact with the magnetic field and build chain clusters. After 60 seconds, the substrate was spun in spin coater at various speed and time. Different viscosity of PVA aqueous solution was also used for optimising the thin film patterns. The thin-film coating was found to be dried only when it was spun at speeds above 700 rpm for 60 seconds, allowing the formed chains to immobilize on the substrate. After optimising the speed, viscosity and spinning time combinations, the array of chains was prepared by spinning the substrate laden with PEG-C-GM-pi-FF having a PVA viscosity of 15.2 mPa as made from 3.3 wt%.

## 3 Results and discussion

During the synthetic preparation of the PEG-C-GM-pi-FF emulsion, several characterization methods were conducted. These methods included the measurement of droplet size, zeta potential, UV-visible absorption, thermal gravimetric analysis, and contact angle. Additionally, intermediate products such as PEG-C-GM, oleic acid-coated magnetite nanoparticles, and ferrofluid were also characterized for relevant properties, including magnetic properties. Furthermore, the morphology of the prepared thin film was discussed. Lastly, Specular Fourier Transformed Infrared Spectroscopy (SR-FTIR) was performed on the patterned thin film to show enhanced absorption (surface enhanced infrared absorption-SIERA) and the correlation of absorption peaks with the film's morphology.

### 3.1 Characterisation of emulsions

#### 3.1.1 Size measurements of nanoparticles and emulsion

**Fig. 3:**
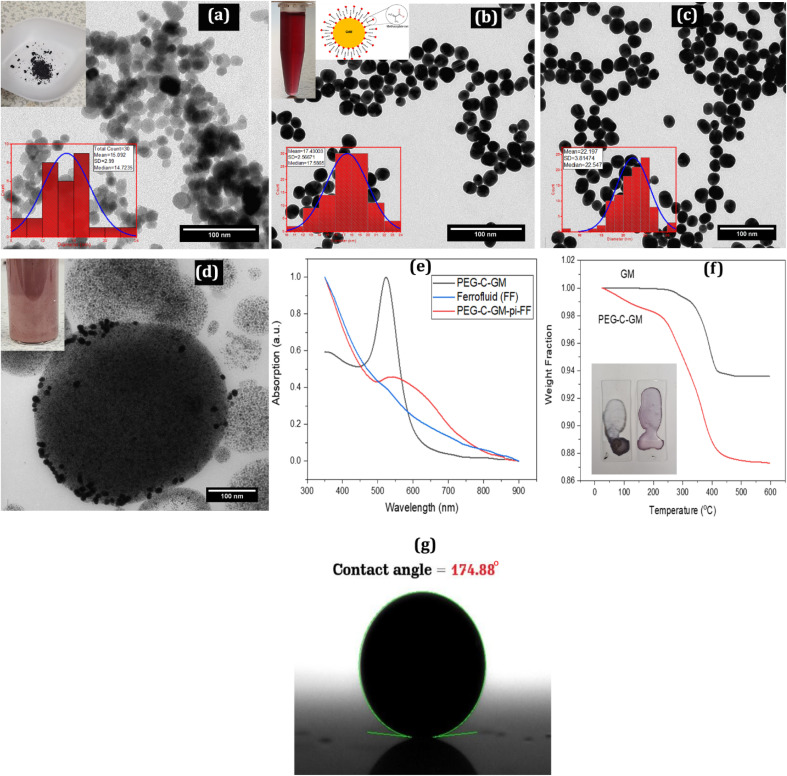
Characterization of colloids, nanoparticles, suspensions, and emulsions prepared using Transmission Electron Microscopy (TEM), contact angle measurements, UV-Vis absorption, and Thermogravimetric Analysis (TGA): (a) TEM image of prepared Fe_3_O_4_ nanoparticles, with an inset showing the size distribution (mean size of ∼15 nm and a standard deviation of 2.99 nm). (b) Size distribution of gold methacrylate (GM) nanoparticles with a mean size of ∼17 nm and a standard deviation of 2.57 nm, with an inset showing an image of the wine-coloured GM solution and methacrylate chains illustration. (c) PEG-coated gold methacrylate nanoparticles, with an inset showing the size distribution (mean size of ∼22 nm and a standard deviation of 3.8 nm). (d) Dried PEG-C-GM-pi-FF droplet. (e) UV-Vis spectra of PEG-C-GM, ferrofluid and PEG-C-GM-pi-FF. (f) TGA of GM and PEG-C-GM, with an inset showing the colour of dried droplets. (g) The contact angle (174.88°) for ferrofluid droplets in PEG-C-GM solutions, obtained using Low Bond Axisymmetric Drop Shape Analysis (LBADSA) plug-in of Image J®.

In the characterization of emulsions, the sizes of oleic acid-coated magnetite (OCM), gold methacrylate (GM), PEG 40S coated gold methacrylate nanoparticles, and PEG-C-GM-pi-FF emulsion droplets were estimated using TEM images. The ferrofluid was dispersed and allowed to dry on a TEM copper grid (300 mesh). The oleic-coated magnetite (OCM) NPs, with an average diameter of 15 ± 3 nm as shown in Fig. 3a, were captured on the grid. The oleic acid coating helped to maintain the stability of the NPs and prevent flocculation.

The average hydrodynamic diameter of gold methacrylate (GM) and PEG-C-GM NPs was determined separately using dynamic light scattering (DLS) with the Malvern zeta sizer. The hydrodynamic diameters were found to be 18.6 ± 3.9 nm and 28 ± 7.4 nm, respectively (ESI Fig. S2†). However, the average mean diameters estimated using TEM were 17 ± 2.6 nm for GM NPs (Fig. 3b) and 22 ± 4 nm for PEG-C-GM NPs (Fig. 3c).

The zwitterionic PEG 40S molecules on the GM NPs facilitated their adsorption on the ferrofluid droplets dispersed in the PVA aqueous solution, as observed in the TEM image (Fig. 3d). In the TEM image, the PEG-C-GM NPs appeared as small dark particles on a large droplet of the ferrofluid. The average diameter of the PEG-C-GM-pi-FF emulsion, measured using the Malvern zeta sizer, exhibited a bimodal ferrofluid droplet size distribution. The distribution had two peaks with central mean diameters of 610 ± 240 nm and 170 ± 16 nm, respectively (ESI Fig. S4†).

To narrow the entire size distribution, centrifugal action was applied to the PEG-C-GM-pi-FF droplets, segregating larger droplets from smaller ones within a specific time frame. A total of 70 000 revolutions were required to achieve a size distribution of approximately 220 ± 50 nm (see ESI, Fig. S*5*†).

#### 3.1.2 Zeta potential

Zeta potential of deionised water (DI), PVA in DI, PEG-C-GM in aqueous PVA solution and PEG-C-GM-pi-FF in PVA solution were measured and presented. The attachment of methacrylic acid molecules led to an increased negative charge on the surface of gold nanoparticles, with a value of −29.8 mV (ESI Fig. S3†), at a pH of 5.6, which is lower than that of gold-acrylate.^52^ However, the zwitterionic PEG 40S coating reduced the charge on the gold NPs by acting as a grafting layer.^53^ However, the zwitterionic PEG 40S coating reduced the charge on gold NPs by acting as a grafting layer.^53^ The PEG-C-GM nanoparticle-stabilized pickering ferrofluid emulsion exhibited a lower electronegativity due to the low zeta potential of the ferrofluid in the PVA aqueous solution. Based on observation that the ferrofluid emulsion was solely stabilized through the adsorption of gold nanoparticles, it can be assumed that the overall charge depended on the number of NPs attached to the surface of the ferrofluid droplets.^54^ Furthermore, the negative zeta potential of PEG-C-GM-pi-FF in the low-zeta potential PVA aqueous solution confirmed the adsorption of gold nanoparticles on the oil droplet. Additionally, the oil droplets were large enough to reduce the overall surface area and zeta potential. The PEG coating, acting as a layer of water-swollen gel, generated supplementary steric repulsions between gold NPs.^53^ Aqueous PVA exhibits low electronegativity, which makes it suitable for working with pickering emulsion. In emulsion, two electronegative charges repel each other, eliminating the possibility of chemical bond formation that could potentially distort the creation of droplet dipole–dipole chains when an external magnetic field is introduced.

ESI Fig. S3† displays the zeta potentials of PEG-capped and uncapped gold methacrylate NPs with the zwitterionic characteristic of PEG.

#### 3.1.3 UV-visible absorption spectroscopy of emulsions

The UV-visible absorption spectrum of 5 μL of ferrofluid redispersed in 20 mL of *cis*-cyclooctene was obtained. The resulting spectrum in Fig. 3e matches that obtained in a previous report.^55^ UV-visible absorption spectra were acquired for PEG-C-GM (Fig. 3e). The surface plasmon vibration of Au colloids appeared at 528 nm wavelength, which is characteristic of spherical Au NPs.^52^ As seen in the Fig. 3b, the prepared GM colloids showed a typical red colour of gold.^52,56^ The methacrylate ion adsorbed on the gold nanoparticles maintains the charge around the particles through hydrophobic–tail interactions, as shown in the schematic in Fig. 3b and prevents flocculation. The UV-visible absorption spectrum of the PEG-C-GM-pi-FF emulsion (Fig. 3e) consisted of a broad absorption band spanning from range 515 nm to 610 nm. It is similar in terms of attaching gold nanoparticles to an iron oxide particle core, as presented in previous works.^57,58^

#### 3.1.4 Thermal gravimetric analysis and contact angle

Thermal Gravimetric Analysis (TGA) revealed that the thermal degradation of methacrylate on the GM surface initiated at approximately 270 °C and completed at 420 °C, resulting in a 6.5% loss of mass (see Fig. 3f). For PEG-C-GM, there was an initial gentle decline in mass observed between 30 °C and 260 °C, followed by a steep decline as the temperature rose to 420 °C, showing a total mass reduction of approximately 12.8%. The number of methacrylate molecules per gold nanoparticle surface was calculated to be 25 665 molecules (see ESI S1.12† for the calculation step).^59^

Images of the droplets for different PVA concentrations were analysed using the contact angle plugin of ImageJ® software.^60^ The contact angle between the glass slide and the PVA droplet increased with increasing PVA concentration (ESI Fig. S6c†). A typical image in ESI Fig. S6a and b† shows a contact angle of 16.9° for a 3.3 wt% solution, confirming the good adhesion of the PVA solution to the substrate. The interfacial tension between PEG-C-GM-pi-FF and the glass slide in DI water, based on the (LBADSA) plugin of ImageJ®,^61^ gave a contact angle of 174.88°. The image scale was set to 316 pixels per mm (Fig. 3g).

#### 3.1.5 Magnetic hysteresis

A superconducting quantum interference device (SQUID) was used to measure the magnetic moments. As seen in ESI Fig. S1A,† the saturation magnetization (*M*_d_) for bulk Fe_3_O_4_ is 446 kA m^−1^, and the saturation magnetization (*M*_s_) for magnetite (Fe_3_O_4_) and OCM nanoparticles are 374.8 kA m^−1^ and 219.9 kA m^−1^, respectively. The magnetic susceptibility (*X*_iL_), which is the initial slope of the curve intercepting at zero magnetic field for Fe_3_O_4_ and OCM, is 8.6 and 1.5, respectively. The coercivity of Fe_3_O_4_ nanoparticles and OCM from ESI Fig. S1B and C† are 1.8 kA m^−1^ and 1.3 kA m^−1^, respectively. The obtained experimental data were used to determine the effective diameter and standard deviation of the tested materials using the model^62,63^ (see ESI S1.8 and Table S1†).

The effective diameter of 13.7 ± 2.94 nm estimated using this method was similar in terms of diameter and standard deviation to the one obtained from the TEM micrograph of OCM in Fig. 3a. The difference between the estimated diameters obtained using the two methods could be attributed to the coating layer (oleic acid), which reduced the effective magnetization of magnetite (Fe_3_O_4_). On the other hand, the estimated diameter (14 ± 3 nm) of the magnetite NPs was much closer to the average diameter obtained by TEM micrograph. The saturation magnetization for the ferrofluid (OCM dispersed in *cis*-cyclooctene) obtained was 29.5 kA m^−1^. This yielded a magnetization ratio between the OCM particles and ferrofluid of 0.06.

### 3.2 Patterned thin film characteristics

#### 3.2.1 Morphology of thin film

**Fig. 4:**
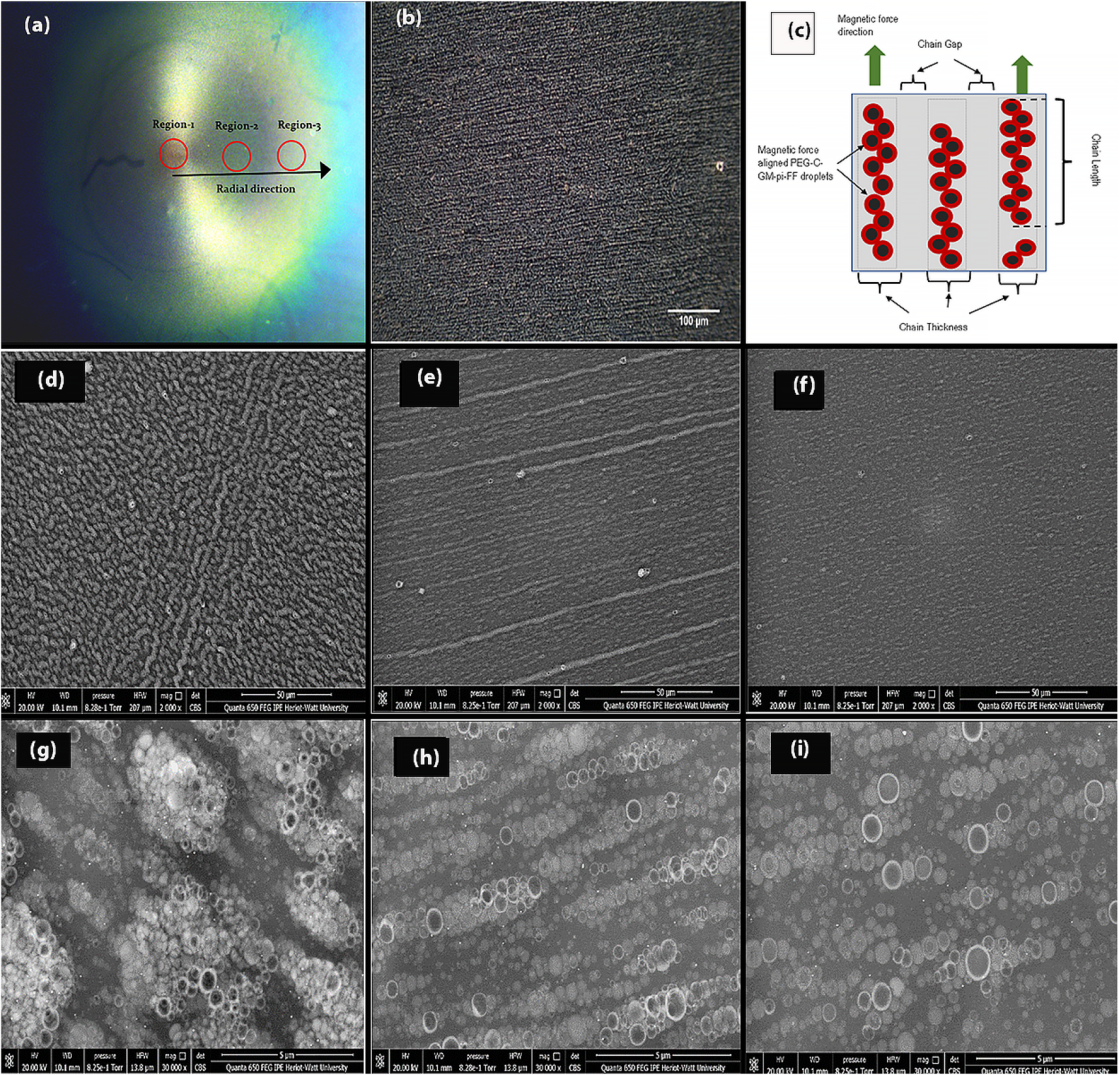
(a) Shows a PEG-C-GM-pi-FF-PVA thin film on a glass slide prepared using MSCDS. The film is divided into three regions, labelled region 1 (*r* = 0 mm), region 2 (*r* = 4 mm), and region 3 (*r* = 7 mm), where “*r*” represents the radial location in millimetres from the centre of the film where the magnet was located. (b) Is an example of a dark field image of arrays of PEG-C-GM-pi-FF obtained using an Olympus BX41 microscope with a 20× 0.4 NA M-plan objective lens. (c) Illustrates the 1D pattern morphology, showing chain length (CL), chain thickness (CT), and chain gap (CG). (d and g) Are scanning electron microscope (SEM) images of the thin film at the centre of the substrate (*r* = 0 mm) at various resolutions (resolution 17 pixels and 1820 per μm, respectively), showing dense clusters of PEG-C-GM-pi-FF. (e and h) Are SEM images of the thin film at *r* = 4 mm, displaying longer chain structures with an average of 4 droplets thickness, (resolution 17 pixels and 1820 per μm, respectively). Lastly, (f and i) Are SEM images of the thin film at *r* = 7 mm, showing shorter chains with a maximum of 2 droplets thickness (resolution 17 pixels and 1820 per μm, respectively).

MSCDS processing of PEG-C-GM-pi-FF in PVA yielded a thin film on a glass slide, as shown in Fig. 4a. Optical and scanning electron microscopy of the thin film showed arrays of parallel chains of PEG-C-GM-pi-FF on the substrate with varying spatial densities. Therefore, the image shown in Fig. 4a was divided into three regions. A typical dark-field image of the thin film (region 2) coated with PEG-C-GM-pi-FF in PVA is shown in Fig. 4b.

In all cases, drying started from the outer edges of the substrate and was directed inward, generating a circular pattern whose diameter decreased with increasing spinning time. The film in the region of interest was divided into three regions, as shown in Fig. 4a.

Fig. 4g–i show the corresponding chain arrays at higher resolution (1820 pixels per μm), where emulsion droplets are visible with white rings appearing as corona of the adsorbed gold nanoparticles at the circumference. Due to the high magnetic strength at the centre of the magnet, thicker columns with worm-like or labyrinth-like formats were established. The chain length (CL) of PEG-C-GM-pi-FF was found to be shorter and densely packed at the centre (Fig. 4d) of the film, but it increased in length at a location away from the centre (Fig. 4e). However, chain formation was no longer visible at a location far away from the centre of the rotation of the film according to the SEM image (Fig. 4f), and that location was not included in the image analysis. These chains were separated by a small or negligible gap. To formally represent the geometrical features of these chains on the substrate, the chain gap (CG), chain length (CL), and chain thickness (CT) are defined in Fig. 4c. Some thin-film nanostructures possess two-fold symmetries indicating a long-range ordering of particles.^64^ CL, CT, and CG were quantified using image analysis (ESI Fig. S7†) to estimate the effect of the geometrical features of the patterned thin film. Further details related to the image analysis are provided in ESI S-1.14 and S-1.15.†

### 3.3 Specular reflectance Fourier transformed infrared spectroscopy (SR-FTIR)

The infrared vibration signals from the PVA adsorbed on the PEG-C-GM-pi-FF arrays in thin film (prepared using 15.2 mPa as viscosity solution at a spin speed of 2500 rpm) were observed. Please refer to ESI S1.7† for the description of the SR-FTIR method.

**Fig. 5:**
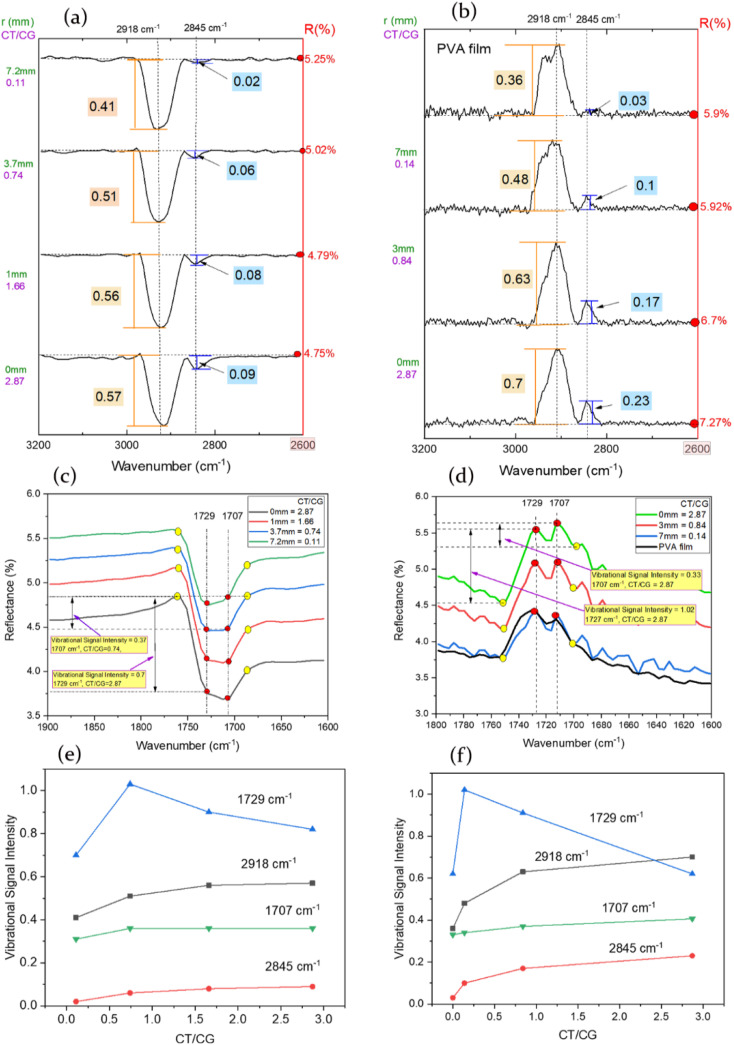
The figure presents the results of specular reflectance FTIR spectroscopy of PEG-C-GM-pi-FF chains on a glass slide at different radial distances from the centre of the thin film, where a magnet was placed. The CT/CG ratio is highest at the centre of the film (*r* = 0 mm) and lowest at the outermost location on the film (*r* = 7.2 mm): using 2600 cm^−1^ as the reference reflectance intensity point (*R*%), (a) displays the variation in the signal intensity of the –CH bond vibrations (2918 cm^−1^ and 2845 cm^−1^) at grazing angle of 20°, while (b) is for 45° grazing angle. (c) Depicts the change in vibrational intensity of the CO bond (1729 cm^−1^ and 1707 cm^−1^) at grazing angles of 20° and (d) for 45° grazing angle. Plots (e) illustrate the relationship between the intensity of vibrational peaks and the CT/CG values at grazing angle of 20° and (f) 45° grazing angle.

The complete FTIR spectrum at angles of incidence (20°, 45° and 82°) can be found in ESI Fig. S8,† covering the range of 4000 cm^−1^ to 650 cm^−1^. To process the different spectra, baseline corrections were performed using Origin® software. For the selected range of 3200 cm^−1^ to 2600 cm^−1^, the reflectance baseline was established at 2600 cm^−1^ for 20° (Fig. 5a) and 45° (Fig. 5b). The baseline for 82° spectrum can be seen in ESI Fig. S9.† The range from 1900 cm^−1^ to 1600 cm^−1^ did not require a baseline reference as the vibrational intensities were sufficiently significant for differential analysis. This can be observed in 20° (Fig. 5c) and 45° (Fig. 5d) reflection spectra, while the reflection spectrum for 82° can be found in ESI Fig. S9.†

#### 3.3.1 Peaks in range – 3200 cm^−1^ to 2600 cm^−1^

The broad absorption band observed at 3600–3000 cm^−1^ (ESI Fig. S8†) corresponds to the OH stretching vibrations in PVA molecules. Additionally, the vibration between 2923–2900 cm^−1^ can be attributed to the asymmetric stretching of the –CH_2_ group in the PVA molecules within the prepared arrays of PEG-C-GM-pi-FF chains on the glass substrate.^65,66^ In the Fig. 5a and b, it can be observed that the intensity of the symmetric stretching vibrational mode at 2846 cm^−1^ associated with the –CH_2_,^67^ increased relative to the asymmetric vibrational intensity (∼2918 cm^−1^) and in relation to the radial distance for both grazing angles. The vibrational intensity of both –CH_2_ peaks was highest in the spectra obtained from the centre of the film (*r* = 0 mm) and decreased as the radial distance from the centre (*r* = 7.2 mm) for both grazing angles. Thicker chains with a higher concentration of gold NPs are likely to contribute to the increase in vibrational peak intensity. This observation is consistent with previous findings that demonstrate an increase in vibrational intensity of both peaks (2918 cm^−1^ and 2845 cm^−1^) in relation to the concentration of gold NPs within the PVA gold composite.^68^ The interaction between gold and iron oxide NPs in the arrays of PEG-C-GM-pi-FF chains potentially enhances charge transfer between all NPs to propagate the electromagnetic field throughout the PEG-C-GM-pi-FF chains in the thin film.^69^

#### 3.3.2 Peaks in range – 1900 cm^−1^ to 1600 cm^−1^

In Fig. 5c and d, the FTIR spectrum of the PVA adsorbed on the PEG-C-GM-pi-FF chain arrays revealed the presence of vibrational modes at 1367 cm^−1^ and 1409 cm^−1.^ These modes are associated with –OH bending in C–H wagging or –CH_3_ stretching and C–H deformation, respectively. The vibrational mode observed at 1707 cm^−1^ can be attributed C=O vibrations,^66,67^ while the ∼1729 cm^−1^ was ascribed to C=O vibration associated with acetate molecules within the PVA (88% hydrolysed).^70^ At the centre of the substrate (*r* = 0), there was a relative increase in the intensity of the C=O (1707 cm^−1^) vibrational mode compared to the C=O (1729 cm^−1^) vibrational mode. However, the intensity of both peaks decreased in all spectra obtained from locations on the thin film at a high radial distance (*r* > 7 mm from the centre of the film). The difference in vibration signals was related to the chain thickness-chain gap ratio (CT/CG) and chain thickness (CT) for angles of incidence of 20° and 45°. The spectra for the 82° angle are presented in ESI S1.16.†

#### 3.3.3 Surface morphology dependent surface enhanced infrared absorption

The nano-printed gold grating enhances the vibrational modes of the monolayer of the chemical analyte. Leading to local field enhancement, as demonstrated by the Specular Reflectance Fourier Transform Infrared (SR-FTIR) technique.^71^ The vibrational band frequencies of the adsorbed molecules scale with the size and period of the grating,^72^ resulting in the amplification of the local electric field. To investigate the effect of chain thickness and gaps in the fabricated thin film on the infrared (IR) absorption of adsorbed PVA molecules, the peaks of SR-FTIR spectra obtained at angles of 45°, 20°, and 82° were correlated with the CT/CG ratio. The same locations on the thin films containing chains of PEG-C-GM-pi-FF adsorbed with PVA were used to acquire the SR-FTIR spectra and images for deriving the CT/CG ratio. In Fig. 5a–d, the vibrational mode intensities of the local minima points (yellow circles) were subtracted from the maximum vibrational mode intensities (red circles) associated with each spectral position to determine the surface-enhanced absorption signals. Subsequently, the obtained vibrational signals were correlated with each CT/CG ratio (Fig. 5e and f). It should be noted that the corresponding values of *r* (mm), CT (μm), and CT/CG were correlated (see ESI Table S5†); therefore, these terms are used interchangeably in this section.

The relationship between CT/CG and the vibrational signals of PVA–CH_2_–symmetric and PVA–CH_2_– asymmetric was found to be nonlinear at all angles of incidence (20°, 45°). The –CH_2_ asymmetric and symmetric vibration resonance modes ascribed to the methylene group present in PVA were of the same intensity as those at *r* = 7 mm (Fig. 5a and b). At 20° incidence, the vibration signals of the –CH_2_-asymmetric band increased with the CT/CG ratio (Fig. 5e). A gentle increase in vibrational intensity up to the highest CT/CG ratio at 2845 cm^−1^ was observed. The –CH_2_ symmetric and asymmetric vibrational signals increased by 350% and 39%, respectively, with an increase in the CT/CG ratio of 170%, and a similar increasing trend for chain thickness (ESI Table S5†) was also observed. The charge interaction between iron oxide-gold NPs would have increased with an increase in the CT/CG values. The increased charge transfer at high CT/CG values may have increased the vibrational signals. The increase in the PVA–CH_2_–symmetric and asymmetric vibrational signals was more pronounced at 45° incidence angle by 667% and 94%, respectively (Fig. 5f), because of the increase in the optical beam area and path within the patterned thin film. The increase in the vibrational signal intensity was 58% at 20° angle (ESI Fig. S9a†).

The reduction in the gaps between PEG-C-GM-pi-FF chains combined with increased chain thickness, also provided a dense hot spot volume and surface sites for the interface between PEG-C-GM-pi-FF droplets, leading to a broadband local field enhancement.^67^ This broad band field enhancement could play a role in enhancing the absorption of the spectrally distributed vibrational bands.^71^ The gold-iron oxide NPs interface provides an enhanced local electric field when in proximity with another, especially where the cluster is denser with little gaps between particles, as seen in some gold assemblies.^69^ Band CA–CO and PVA–CO also responded to the trend of chain thickness for all incident angles, where they had approximately the same vibration signal intensities at both 20° and 45° (Fig. 5e and f). At 20° incident angle, the change in CA–CO and PVA–CO vibrational signals in the spectrum obtained from area on thin film, (where CT values were between 1.01 μm to 2.82 μm) was 16% and 17% respectively. At 45° incident angle, this decreased by 39% for PVA–CO–CO peak and increased by 19% for CA–CO peak under the same range. Meanwhile, at 20°, this decreased by 22% for PVA–CO and decreased by 16% for CA–CO within the same range. This was because of the absorption value at these bands for a single-layer PVA film, which was the same as the value at *r* = 0 mm (Fig. S9b†). However, there was a noticeable decrease in the intensity of the peaks at lower CT/CG values (Fig.S9c†).

#### 3.3.4 Grazing angle optimisation

The increase in the grazing angle improves the interaction of light with the thin film owing to spatial extension but decreases the optical density. While increased spatial extension enhances the vibration signals, decreased optical density reduces the detection sensitivity. The vibrational signal intensities were highest in the spectra obtained at 45° grazing angle (Fig. 5f) and lowest at 82° grazing (Fig. S9c†). At 45°, the optical density and spatial coverage of the PEG-C-GM-pi-FF chains were optimized to obtain the highest vibrational signals. These angles may be particular to the geometry of the thin film generated in this study because the greater grazing angle on the chains with a given height might cast a shadow of one strip on to the other, limiting the overall exposure of light on the material.^70^ Another possibility is that incident wave backscattering increases at greater grazing angles.^73^ Therefore, the optical waves interacted poorly with the thin-film pattern and glanced at the highest grazing angle. Overall, the magnetically directed self-assembly established near and far-field interactions through good dispersion, providing tunability.^73–75^

## 4 Conclusion

This study demonstrates the utilization of magnetic directed spin-coating self-assembly (MDSCSA) to fabricate an optically sensitive film consisting of periodic nanoarrays of gold nanoparticle-stabilized pickering ferrofluid emulsion chains in PVA on a silica glass substrate. The variation in the chain thickness (CT) and gaps (CG) between the chains enhances the vibrational signal of the CH_2_ infrared absorption bands. The enhancement in the CT/CG ratio from 0.11 to 2.87 resulted in a 667% increase in the infrared vibration signal of the CH_2_ (2918 cm^−1^) bond (in comparison to 2845 cm^−1^) at 45° beam incidence, while the vibrational signals dropped at 20° angle. These results suggest the potential of using gold nanoparticles with Fe_3_O_4_ to create tuneable infrared resonant peak strips for surface-enhanced infrared spectroscopy. The structure of this film combines both far- and near-field effects to locally enhance the charge and vibration of the attached molecules. The variability in the resolution of a single thin film allows for greater flexibility in identifying and comprehending species traits under different CT/CG patterns. Future work will involve investigating the minimum quantity of gold nanoparticles required in relation to Fe_3_O_4_ nanoparticles to establish both near-UV and near-infrared plasmon effects.

## Data availability

A open access link to the raw data will be provided after the acceptance of the manuscript.

## Conflicts of interest

There are no conflicts to declare.

## Supplementary Material

RA-013-D3RA01369C-s001

## References

[cit1] Henares T. G., Mizutani F., Hisamoto H. (2008). Current development in microfluidic immunosensing chip. Anal. Chim. Acta.

[cit2] Dehghani S., Nosrati R., Yousefi M., Nezami A., Soltani F., Taghdisi S. M., Abnous K., Alibolandi M., Ramezani M. (2018). Aptamer-based biosensors and nanosensors for the detection of vascular endothelial growth factor (VEGF): A review. Biosens. Bioelectron..

[cit3] Robert J. W., Wei H., Cheryl Wong P. F., Ai Leen K., Richard S. G., Christopher E. M. (2009). Formation and Properties of Magnetic Chains for 100nm nanoparticles used in separation of molecules and cells. J. Magn. Mater..

[cit4] Lu Z., Yadong Y. (2012). Colloidal nanoparticle clusters: functional materials by design. Chem. Soc. Rev..

[cit5] Zhijie Y., Jingjing W., Konrad G., Myung-Geun S., Bartosz G. (2017). Interference-like patterns of static magnetic fields imprinted into polymer/nanoparticle composites. Naturecomms.

[cit6] Li Z. , Bradely J. N. and Lixin D., Magnetic-Field-Based Self-Assembly, Springer, Dordrecht, 2012

[cit7] Jianping G., Yongxing H., Yadong Y. (2007). Highly tunable suparamagnetic colloidal photonic crystals. Angew. Chem., Int. Ed. Engl..

[cit8] Ge J., He L., Goebi J., Yin Y. (2009). Assembly of magnetically tunable photonic crystals in non-polar solvents. J. Am. Chem. Soc..

[cit9] Henderson J., Shi J., Cakmaktepeand T S. (2012). Crawford Pattern transfer nanomanufacturing using magnetic recording for programmed nanoparticle assembly. Nanotechnology.

[cit10] Kuo-Feng H., Jung-Wwei L., CHeng-Yu H., Liang-Wei W., Yen-Chun H., Wei-Chih W., Mu-Tung C., Shen-Chuan L., Jun Y., Hsiu-Hau l., Chih-Huang L. (2015). Magnetic patterning: local manipulation of the intergranular exchange coupling via grain boundary engineering. Sci. Rep..

[cit11] Herb H., Christopher J. B., William E. L. (2004). Ferrofluid-based microchip pump and valve. Sens. Actuators, B.

[cit12] Philip J., Kumar T. J., Kalyanasundaram P., Raj B. (2003). Meas. Sci. Technol..

[cit13] Sahu N., Parija B., Panigrahi S. (2009). Fundamental understanding and modeling of spin coating process: A review. Indian J. Phys..

[cit14] Le H., Wang M., Zhang Q., Lu Y., Yadong Y. (2013). Magnetic Assembly and patterning of General Nanoscale Materials through Nonmagnetic Templates. Nano Lett..

[cit15] Yellen B. B., Ondrej H., Gary F. (2005). Arranging matter by magnetic nanoparticle assembler. PNAS.

[cit16] Srinivasan G., Sreenivasulu G., Benoit C., Petrov V. M., Chavez F. (2015). J. Appl. Phys..

[cit17] Fiabane J., Prentice P., Pancholi K. (2016). High yielding microbubble production method. BioMed Res. Int..

[cit18] Claire A., Mohamed B., Nicolas T., Elias F., Florence A., Nicolas H. (2019). Pickering emulsions: Preparation processes, key parameters governing their properties and potential for pharmaceutical applications. J. Controlled Release.

[cit19] Kurdak C., Kim J., Farina L., Lewis K. M., Bai X., Rowe M., Matzger A. (2003). Au Nanoparticle Clusters: A New System to Model Hopping Conduction. Turk. J. Phys..

[cit20] Luchao D., Guirong X. S. Z., Akihiro F. (2020). Plasmon induced charge transfer mechanism in gold-TiO2 nanoparticle systems: The size effect of gold nanoparticle. J. Appl. Phys..

[cit21] Bowei X. L. M., Junming Z., Linhua L. (2019). Dependent absorption property of nanoparticle clusters: an investigation of the competing effects in the near field. Opt. Exp..

[cit22] Fan X., Zheng W., Singh D. J. (2014). Light scattering and surface plasmons on small spherical particles. Light: Sci. Appl..

[cit23] Jingyu S., Feng T., Jing L., Mo Y. (2015). Nanoparticle based fluorescence resonance energy transfer (FRET) for biosensing applications. J. Mater. Chem. B.

[cit24] Eunkeu O., Alan L. H., Andrew S., Alexander E., Marc C., Kimihiro S., Konrad B., Ramasis G., Fredrik K. F., Igor L. M. (2016). Energy Transfer Sensitization of Luminescent Gold Nanoclusters: More than Just the Classical Förster Mechanism, Scientific Reports. Nature.

[cit25] George C., Genovese A., Qiao F., Korobchevskaya K., Comin A., Falqui A., Marras S., Roig A., Zhang Y., Krahne R., Manna L. (2011). Optical and electrical properties of colloidal (spherical Au)-(spinel ferrite nanorod) heterostructures. Nanoscale.

[cit26] Comin A., Korobchevskaya K., George C., Diaspro A., Manna L. (2012). Plasmon bleaching dynamics in colloidal gold–iron oxide nanocrystal heterodimers. Nano Lett..

[cit27] Korobchevskaya K., George C., Diaspro A., Manna L., Cingolani R., Comin A. (2011). Ultrafast carrier dynamics in gold/iron-oxide nanocrystal heterodimers. Appl. Phys. Lett..

[cit28] Robert J. W., Wei H., Wong P. F. C., Leen K. A., Richard G., Christopher M. E. (2009). Formation and Properties of Magnetic Chains for 100nm nanoparticles used in separation of molecules and cells. J. Magn. Magn. Mater..

[cit29] Alexey O., Andrey Z. (2020). Chain formation and Phase separation in ferrofluid: The influence of viscous properties. Materials.

[cit30] Ryan S., Jason B., Kevin C., Alamgir K. (2011). Imaging magnetic flux lines with iron oxide nanoparticles using a fossilized liquid assembly. Soft Matter.

[cit31] Zhihan Z., Guojun L., Dehui H. (2009). Coating and Structural Locking of Dipolar Chains of Cobalt Nanoparticles. ACS Nano.

[cit32] Samir M., Damien L. R., Marie-Charlotte A., Joël L., Véronique D. (2018). Arrays of high aspect ratio magnetic microstructures for large trapping throughput in lab-on-chip systems. Microfluid. Nanofluid..

[cit33] Leal-Calderon F., Stora T., Monval M. O., Poulin P., Bibette J. (1994). Direct measurement of colloidal forces. Phys. Rev. Lett..

[cit34] Leal-Calderon F. , Veronique S. and Jerome B., Emulsion science: Basic principles, Springer Science and Business Media, New York, 2007

[cit35] Abargues R., Rodriguez-Canto P. J., Albert S., Suarez I., Martínez-Pastor J. P. (2014). Plasmonic optical sensors printed from Ag–PVA nanoinks. J. Mater. Chem. C.

[cit36] Conceicao T., Scharnagl N., Blawert C., Dietzel W., Kainer K. (2010). Corrosion protection of magnesium alloy AZ31 sheets by spin coating process with poly(ether imide). Corros. Sci..

[cit37] Walheim S., Schaffer E., Mlynek J., Steiner U. (1999). Nanophase-separated polymer films as high-performance antireflection coatings. Science.

[cit38] Sirringhaus H., Nir T., Richard H. F. (1998). Integrated optoelectronics devices based on conjugate polymers. Science.

[cit39] Eaton K., Douglas P. (2002). Effect of humidity on the response characteristics of luminescent PtOEP thin film optical oxygen sensors. Sens. and Actuators B.

[cit40] Mingsheng W., Chuanbo G., Le H., Qipeng L., Jinzhong Z., Chi T., Zorba S., Yadong Y. (2013). Magnetic Tuning Plasmonic Excitation of Gold Nanorods. J. Am. Chem. Soc..

[cit41] Zhang H., Kin-Hung F., Jürgen H., Che Ting C., Dayang W. (2008). Controlled chainlike agglomeration of charged gold nanoparticles via a deliberate interaction balance. J. Phys. Chem. C.

[cit42] Chen H., Shao L., Li Q., Wang J. (2013). Gold nanorods and their plasmonic properties. Chem. Soc. Rev..

[cit43] Ozinand G. A., Yang S. M. (2001). The Race for the Photonic Chip: Colloidal Crystal Assembly in Silicon Wafers. Adv. Funct. Mater..

[cit44] Banik M., Mukherjee R. (2018). Fabrication of Ordered 2D Colloidal Crystals on Flat Patterned Substrates by Spin Coating. ACS Omega.

[cit45] Han G. S., Kim J., Bae S., Han S., Kim Y. J., Gong O. Y., Lee P., Ko M. J., Jung M. S. (2019). Spin-Coating Process for 10 cm × 10 cm Perovskite Solar Modules Enabled by Self-Assembly of SnO_2_ Nanocolloids. ACS Energy Lett..

[cit46] Qin D., Xia Y., Whitesides G. M. (2010). Soft lithography for micro- and nanoscale patterning. Nat. Protoc..

[cit47] Zhao X.-M., Xia Y., Whitesides G. M. (1997). Soft lithographic methods for nano-fabrication. J. Mater. Chem..

[cit48] Rogers J. A., Nuzzo R. (2005). Recent Progress in soft Lithography. Mater. Today.

[cit49] Lai C. W., Low F. W., Tai M. F., Hamid S. B. A. (2017). Iron oxide nanoparticles decorated Oleic acid for high colloidal stability. Adv. Polym. Technol..

[cit50] Okpozo P., Pancholi K. (2023). Study of spatial organisation of magnetic field directed gold-pickering-ferrofluid-nanoemulsion in spin coated film. Hyb. Adv..

[cit51] Berger P., Adelman N. B., Beckman K. J., Campbell D. J., Ellis A. B., Lisensky G. C. (1999). Preparation and properties of an aqueous ferrofluid. J. Chem. Educ..

[cit52] Hussain I., Brust M., Papworth A. J., Cooper A. I. (2003). Preparation of Acrylate-Stabilized Gold and Silver Hydrosols and Gold-Polymer Composite Films. Langmuir.

[cit53] Wang Y., Enrico J., Ono T., Maeki M., Tokeshi M., Isono T., Tajima K., Satoh T., Sato S., Miura Y., Yamamoto T. (2020). Enhanced dispersion stability of gold nanoparticles by the physisorption of cyclic poly (ethylene glycol). Nat. Commun..

[cit54] Hu M., Du X., Liu G., Huang Y., Liu Z., Sun S., Li Y. (2022). Oppositely charged pickering emulsion co-stabilized by chitin nanoparticles and fucoidan: Influence of environmental stresses on stability and antioxidant activity. Foods.

[cit55] Liu H., Hou P., Zhang W., Kim Y. K., Wu J. (2010). The synthesis and characterization of polymer-coated FeAu multifunctional nanoparticles. Nanotechnology.

[cit56] Frens G. (1973). Controlled Nucleation for the Regulation of the Particle Size in Monodisperse Gold Suspensions. Nature.

[cit57] Reguera J., de Aberasturi D. J., Winckelmans N., Langer J., Bals S., Liz-Marzán L. M. (2016). Synthesis of Janus plasmonic–magnetic, star–sphere nanoparticles, and their application in SERS detection. Faraday Discuss..

[cit58] Nguyen T., Mammeri F., Ammar S. (2018). Iron Oxide and Gold Mased Magneto-Plasmonic nanostructures for Medical Applications: A Review. Nanomaterials.

[cit59] Bajaj M., Wangoo N., Jainn D. V. S., Sharma R. K. (2020). Quantification of adsorbed and dangling citrate ions on gold nanoparticle surface using thermogravimetric analysis. Sci. Rep..

[cit60] Brugnara M. , Contact_Angle Image, https://imagej.nih.gov/ij/plugins/contact-angle.html, accessed 27 October 2020

[cit61] Stalder A., Melchior T., Müller M., Sage D., Blu T., Unser M. (2010). Low-bond axisymmetric drop shape analysis for surface tension and contact angle measurements of sessile drops. Colloids Surf., A.

[cit62] Chantrell R., Popplewell J., Charles S. (1978). Measurements of particle size distribution parameters in ferrofluids. IEEE Trans. Magn..

[cit63] O'grady K., El-Hilo M., Chantrell R. (1993). The characterisation of interaction effects in fine particle systems. IEEE Trans. Magn..

[cit64] Giuliani M., Gonzalez-Vinas W., Poduska K. M., Yethira A. (2010). Dynamics of crystals structure formation in spin coated colloidal films. J. Phys. Chem. Lett..

[cit65] Blout E., Karplus R. (1948). The infrared spectrum of polyvinyl alcohol. J. Am. Chem. Soc..

[cit66] Wexler A. (1967). Integrated Intensities of Absorption Bands in Infrared Spectroscopy. Appl. Spectrosc. Rev..

[cit67] Mahendia S., Tomar A. K., Chahal R. P., Goyal P., Kumar S. (2011). Optical and Structural properties of poly(vinyl alcohol) films embedded with citrate-stabilized gold nanoparticles. J. Phys. D: Appl. Phys..

[cit68] Moreno M., Hernandez R., Lopez D. (2010). Crosslinking of poly(vinyl alcohol) using functionalized gold nanoparticles. Eur. Polym. J..

[cit69] Ghosh H., Burgi T. (2017). Mapping Infrared Enhancement around Gold Nanoparticles Using Polyelectrolytes. J. Phys. Chem..

[cit70] Saini I., Sharma A., Chandak N., Aggarwal S., Sharma P. (2018). Ag nanoparticles induced modification in microhardness of polyvinyl alcohol. Adv. Mater. Processes.

[cit71] Maß T., Nguyen V., Schnakenberg U., Taubner T. (2019). Tailoring grating strip widths for optimizing infrared absorption signals of an adsorbed molecular monolayer. Opt. Exp..

[cit72] Wang T., Nguyen V. H., Buchenauer A., Schnakenberg U., Taubner T. (2013). Surface enhanced infrared spectroscopy with gold strip gratings. Opt. Exp..

[cit73] Hawkeye M. (2007). Glancing angle deposition: Fabrication, properties, and applications of micro-and nanostructured thin films. J. Vac. Sci. Technol., A.

[cit74] Gupta R., Gupta P., Footer C. (2022). *et al.*, Tuneable magnetic nanocomposites for remote self-healing. Sci. Rep..

[cit75] Gupta R., Pancholi P. V., Yu X., Gupta L., Stenning G. B. G., Bucknall D., Flynn D., Pancholi K. (2023). Role of interface in optimisation of polyamide-6/Fe3O4 nanocomposite properties suitable for induction heating. Nano-Struct. Nano-Objects.

