# Supplementary material for: Enhancement of infrared absorption through a patterned thin film of magnetic field and spin-coating directed self-assembly of gold nanoparticle stabilised ferrofluid emulsion
Source: RSC Adv. 2023 Aug 10;13(34):23955–66. doi: 10.1039/d3ra01369c (PMC10413183; doi:10.1039/d3ra01369c)
Supplement: RA-013-D3RA01369C-s001 [file RA-013-D3RA01369C-s001.pdf]

Electronic Supplementary Material (ESI) for RSC Advances.
This journal is © The Royal Society of Chemistry 2023

Supplementary Data

Enhancement of Infrared Absorption Through a Patterned Thin Film of Magnetic Field and Spin-Coating Directed Self-Assembly of Gold Nanoparticle Stabilised Ferrofluid Emulsion

Paul Okpozo[1], Yashashchandra Dwivedi[2], Dehong Huo[3]and Ketan Pancholi[1,4]*

[1]School of Engineering, Sir Ian Wood Building, Robert Gordon University, Garthdee, Aberdeen, AB10 7GJ, UK

[2]Physics Department, National Institute of Technology Kurukshetra, Kurukshetra, 136119, India

[3]School of Engineering, Newcastle University, Newcastle, NE1 7RU, UK

[4]Advanced Materials Group, School of Engineering, Robert Gordon University, Aberdeen, UK

*Email:k.pancholi2@rgu.ac.uk

S1.1 Transmission Electron Microscope (gold and iron oxide nanoparticles, Pickering droplets)

JEOL® 1200TEM – 80kV was used capture TEM images of magnetite, gold methacrylate nanoparticles, PEG coated gold methacrylate nanoparticles and Pickering droplets. 5µL of magnetite ferrofluid and PEG-C-GM suspension was deposited on TEM grids and allowed to dry under vacuum at 20°C. As for the Pickering droplet in polymer solution, a microscopic slide was mechanically etched using a hand drill to contain and hold the TEM grid. The solution was spin-coated over the grid and glass slide. The grid was removed and no further action was taken prior to the electron microscope scan.

S1.2 Dynamic light scattering and Zeta potential characterisation

DLS measurements were conducted using a Malvern® Zetasizer Nano ZS instrument at a fixed angle of 173° (backscattering detection) with a He–Ne laser (λ = 633 nm; 4 mW) and digital autocorrelator. The mean particle size was determined by a number plot. Size distribution of centrifuged Pickering droplets was conducted as follows;

- 5µL from centrifuged solution diluted in 10ml of DI water
- 3ml from the mixture poured into a polymethylmethacrylate (PMMA) cuvette.
- Scan settings was set to 14 runs per scan at 20°C operating temperature with cumulative average of three scans.

Zeta potential measurement were conducted for freshly prepared colloidal dispersions and Pickering emulsions by diluting test solution in DI water and injected into Malvern ® DTS1070 disposable cuvettes. 12 Zeta runs at 21°C operating temperature was the setting and the concentration of samples tested are as follows;

- 1.12±0.1µg/L of GM in DI water
- 1.5±0.2µg/L of PEG-C-GM in DI water
- 5µL of PEG-C-GM-Pi-F was diluted in 10ml of water
- Aqueous PVA was further diluted to 0.01wt.% in water
- 0.2mg of Ferrofluid ultrasonicated in 20ml of 1.7wt.% aqueous PVA. 10µL of emulsion diluted in 25ml of water.
- DI water used as base test liquid.

S1.3 Contact angle measurement

In the first part, glass slides were used as the substrate as 5wt.%, 3.33wt.%, 1.7wt.%, and 1.25wt.% PVA liquid concentration were placed close to the edge of the substrate which was over an x, y and z adjustable stage. 8µL of each concentration was for contact angle measurement, while 0.5ml for thickness of fluid above the substrate. In the second part, half of the glass slide length was placed in a square shaped glass chamber (50mm length, 30mm width and 40mm height) filled with 0.1mg/L of GM in DI water as ~400µm Pickering emulsion droplet from rotor stator emulsification process was displaced over the glass substrate. The images were captured using a 2-megapixel infinity CCD coloured camera and 0.7 – 4X magnification zoom lens system.

S1.4 Magnetic Hysteresis measurement of OCM and ferrofluid

This process was carried out using Superconducting quantum interference device (SQUID), a magnetometer device that measures extremely subtle magnetic fields as low as 5 X 10-14Tesla. 1g each of the samples were tested at ambient temperature (300K). The test was carried out on both magnetite particles, Oleic coated magnetite (OCM) and ferrofluid.

S1.5 Optical Microscopy of Thin Film

Images of samples were captured using an Olympus® BX41 Darkfield microscope coupled with Yencam HD II camera, having a 6 megapixels CMOS sensor. Images were magnified using an M-plan Achromat MPLN-BD 50X, NA 0.5 magnification lens. The position of the slides was shifted along one axis while the other axis was kept constant. The positional readings were recorded from the Vernier scale distribution on the microscope stage as snapshots of the samples were taken. The images were analysed using Image J® tool.

S1.6 Scanning Electron Microscopy (SEM)

Dried spin coated samples were tested using a Quanta 650 FEG SEM. A low-pressure vacuum of 0.825 Torr, accelerating voltage for electrons (HV) is 20kV and working distance of 10±1um were used for each point scan depending on the magnification.

S1.7 Specular Reflectance FTIR

Two types of equipment were used for this test. The first was a Thermo Scientific™Nicolet™iN™ 10 infra-red microscope. It was used to conduct both transmission and reflection mode tests on thin film nanostructure on glass side. 50µm^2 aperture was used to observe sample area through a 15X, 0.7 NA objective lens that creates a near normal incidence of beam interaction with surface of sample is a half angle - 20° [1], as it is common to have a slightly off normal incident beam for most commercial equipment [2]. The infra-red scan range was set to read a range from 450 cm^{-1} to 4000 cm^{-1} with number of scans set to 16 as background corrections were taken before any new sample was tested by scanning an uncoated glass slide.

The second was a Agilent® 4100 ExoScan FTIR analyser, scanning spectral range between 5200-650 cm^{-1}, conducting 64 background and sample scans with resolution of 8 cm^{-1}. In this

experiment, 45° and 82° infra-red beam incidence angle from normal were tested for a single layer of coating.

S1.8 Calculations for the nanoparticle diameter

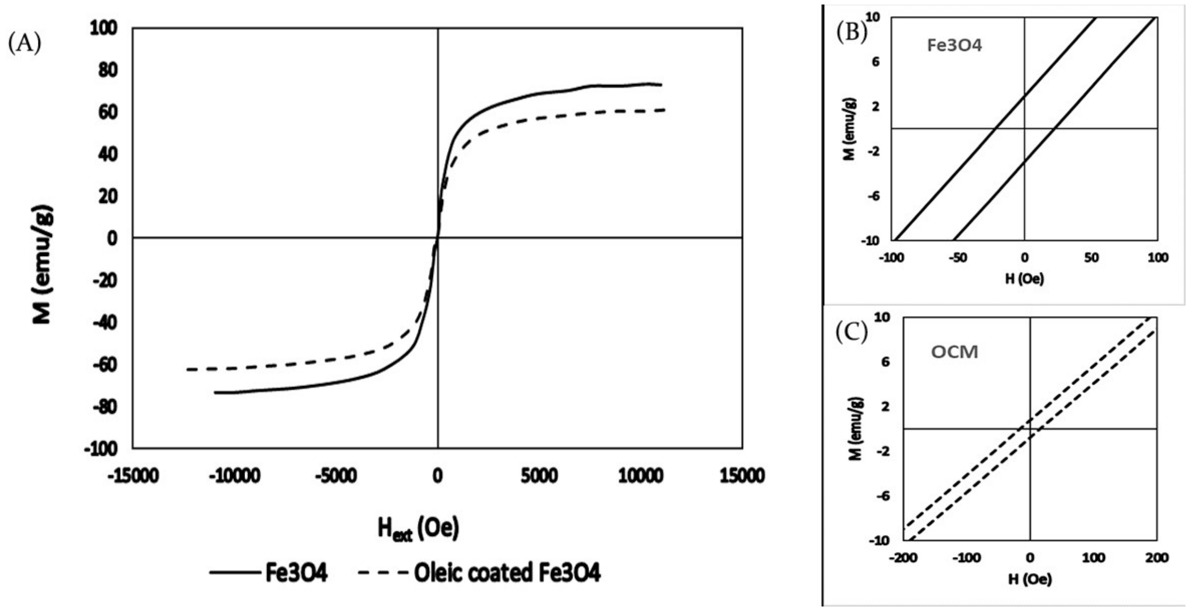

Figure S.1 (A) Magnetic hysteresis for magnetite and Oleic coated magnetite (OCM), (B) and (C) presents a magnified range of the hysteresis curve to obtain coercivity (H_o) for both magnetite and OCM materials respectively.

$$D_m = \left\{\left[\frac{18KT}{\pi M_d \mu_o}\right] \times \sqrt{\left[\frac{\chi_{iL}}{3M_s H_o}\right]}\right\}^{1/3}$$ [eq-S1]

$$\sigma_m = \sqrt{\ln\left(3\chi_i H_o\right)}$$ [eq-S2]

$$\chi_i = \frac{\chi_{iL}}{1 + {}^{1}/_{3}\chi_{iL}}$$ [eq-S3]

Where,

M_d is the saturation of magnetization of bulk material, χ_{iL} is the initial susceptibility from magnetization of test sample, χ_i *is the reduced initial susceptibility* M_s is saturation magnetization of test sample, H_o is the coercivity of the test material, μ_o is the is free space magnetic permeability, given as $4\pi \times 10^{-7}$ N/A^2.

Table-S 1: Summary of results derived from the magnetic hysteresis (Figure 2(h) in main article) and estimated diameter of NPs with associated standard deviation.

**Nanoparticle Material**	**Density (Kg/m^3)**	**X_{Il}**	**X_I**	**Ms(A/m)**	**Ho (A/m)**	**M_d (A/m)**	**D_m (m)**	**σ_m**
Fe_3O_4	5150	8.6	2.2	374755	1753.4	446000	1.41E-08	3.06
OCM	3640	3.16	1.54	219907	1275.2	446000	1.37E-08	2.95

1 Oe = 79.5575 A/m, K_B = 1.381 X 10^{-23}N.m/K, 1 emu/g = 1 Am^2/kg

S1.9 DLS size measurement of GM and PEG-C-GM nanoparticles and Zeta potentials of mixed components

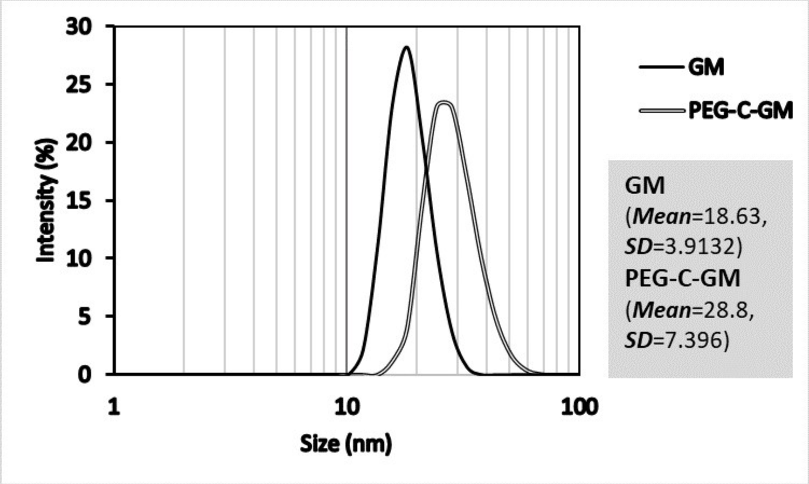

Figure S 2: Dynamic light scattering measurement of gold methacrylate (GM) and PEG-C-GM

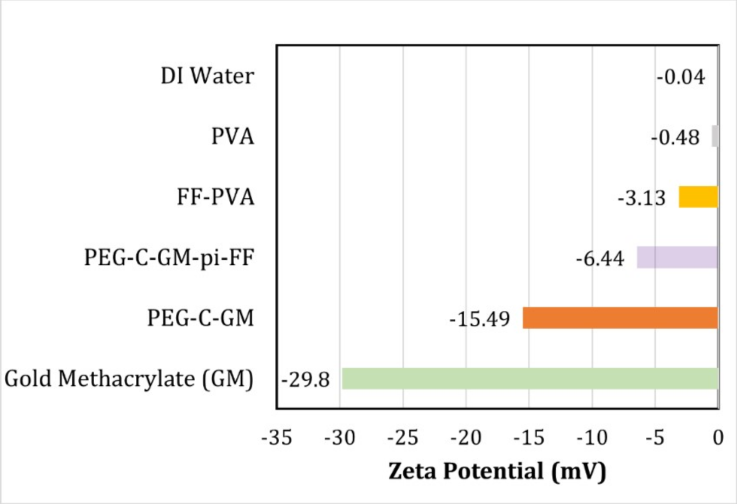

Figure S 3: Presentation of Zeta potential peak values of varying particle systems

S1.10 PEG-C-GM-pi-FF size management by centrifugation

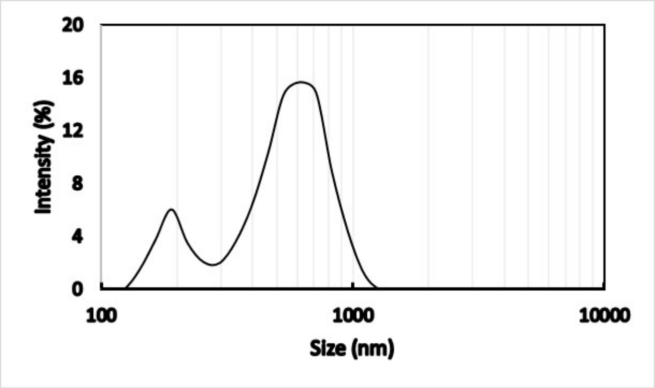

Figure S 4: Spectra of size distribution of ultrasonicated mixture using DLS Malvern Zeta sizer

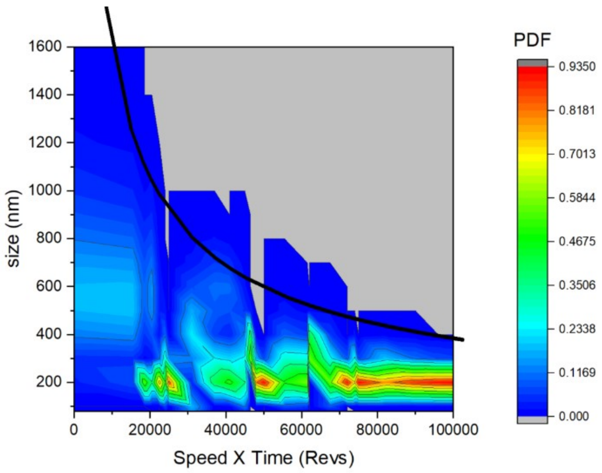

Figure S 5: Trend line fitting within PDF range (0.005 – 0.09) in a 3D surface plot, generating a power curve with decline rate of 0.6, R^2=0.85

S1.11 Polyvinyl alcohol properties (Density and viscosity)

The density of PVA can easily be determined from works done by Mohsen-Nia and Modarress [3], who carried out test for 88,000 Molecular weight (88% hydrolysed) PVA, for the case of operating at 20°C. The viscosity (η) in mPas was determined using a model for PVA with Molecular weight between 86.5-89% [3].

Table-S 2: Calculated values of densities and viscosities calculated using the model [3]

Weight Fraction	Density of PVA (g/ml)	Viscosity of Polymer (mPas)
0.0125	1.005286	1.94
0.0167	1.00639	3.2
0.033	1.010676	15.2
0.05	1.015145	43.2

S1.12 TGA for Gold Methacrylate (GM)

Initially, 17.5mg of GM and 13.5 mg of the PEG-C-GM was placed on a platinum pan. The sample was purged with a nitrogen at 10ml/min flow. The sample was heated for 60 minutes from 22°C to maximum temperature of 600°C at a ramp rate of 10°C/min.

Calculations:

The mass of gold remaining (G_{res}): $0.935855 \times 17.515mg = 16.39151mg$

Volume of gold (G_{vol}) using standard density of gold (ρ = 19.3 g/ml):

$$\frac{0.01639151}{19.3} = 8.493 \times 10^{-4} ml \; or \; 8.493 \times 10^{-10} m^3$$

Volume of gold nanoparticle ($G_{NP(vol)}$) considering average particle diameter (d = 17.43nm):

$$\frac{\pi}{6} \times (17.43 \times 10^{-9})^3 = 2.7726 \times 10^{-24} m^3$$

Number of gold nanoparticles ($G_{NP(i)}$) in residue is:

$$\frac{8.493 \times 10^{-10}}{2.7726 \times 10^{-24}} = 3.0632 \times 10^{14} particles$$

The mass of Methacrylate molecules burnt off from gold nanoparticle (MC_m) is:

$$17.515mg - 16.39151mg = 1.12349mg$$

Number of moles in Methacrylate (MC_n) where molecular mass of Methacrylic acid (MM=86.06 g/mol) is:

$$\frac{1.12349 \times 10^{-3}}{86.06} = 1.3055 \times 10^{-5}\, moles$$

Number of molecules of burnt Methacrylic acid (MC_i) using Avogadro's number (Av = 6.02214 X 10^{23} mol^{-1}):

$$1.3055 \times 10^{-5} \times 6.02214 \times 10^{23} = 7.8619 \times 10^{18} molecules$$

Number of Methacrylic molecules per gold nanoparticle (MC_i/ $G_{NP(i)}$) is

$$\frac{7.8619 \times 10^{18}}{3.0632 \times 10^{14}} = 25665\, {}^{molecules}/_{nanoparticle}$$

S1.13 Contact angle measurement

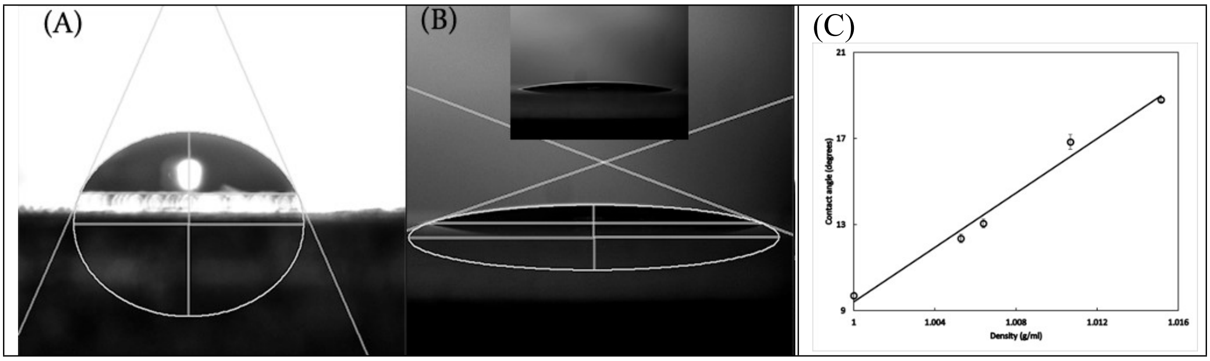

Figure S 6: Contact angle measurements using Contact_angle plugin from Image J® to obtain values for (a) before glass slide treatment (62.25°), (b) after glass slide treatment (16.85°), the insert was the original image, (c) Plot of contact angle against respective PVA densities, using treated glass slide; the fitting line for the plot bears the equation contact angle $(CA) = 631.7^0 - 622.3^0$***, with an R squared of 0.98***

S1.14 Image analysis

The images in Figure S-5 (b, e & h) are binary processed images of Figure S-5 (a, d and g) respectively. While Figure S-5 (c, f & i) are the 3D plot of the scans for the 4 locations (red lines in Figure S-5 b, e & h) separated approximately 50 µm apart. The binary plot distributions appeared thicker for Figure S-5 (c) while least thick for (i). The entire lines are challenging to distinguish, therefore the number of bar divisions greater than zero were counted as droplet, while values at zero taken as gap. The division utilized in this analysis was 167nm/pixel. A brief example of data is shown in Table S-5, where it shows the binary values (>0 and 0) for each 167 nm divisions. This did not actually represent the thickness of chains and chain gaps. Therefore, a simple algorithm was written (presented in S-1.16) to group consistent divisions of white pixel (>0) and black pixel (0) into number of pixels in chain thickness (CntCH) and number of pixels for chain gaps (CntGap)

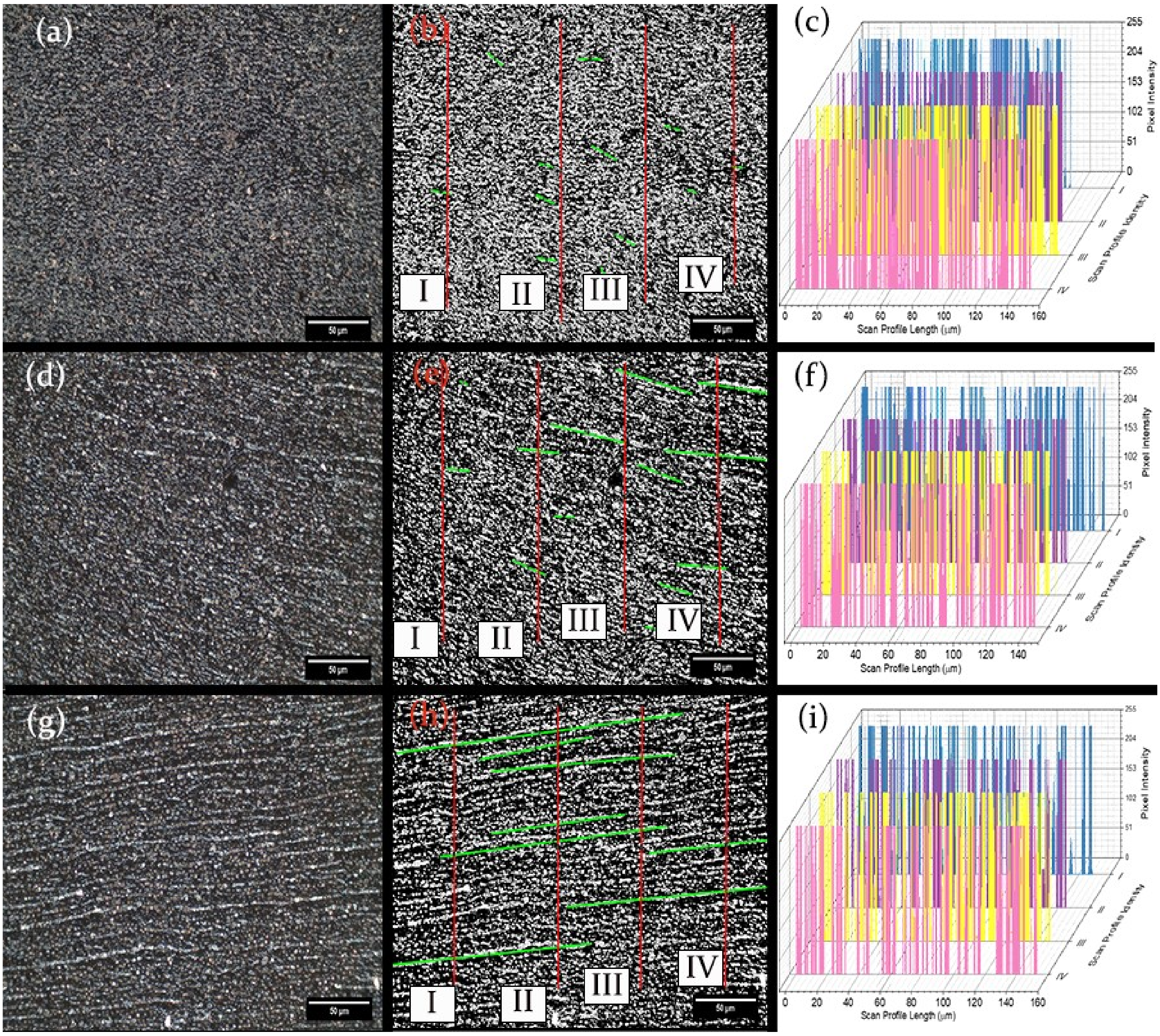

Figure S.7: Average resolution of pitches between chain thickness and gap was determined from plot profile (c, f, i) of images (a,d &g) which was processed into binary construct (b, e & h). The green lines are for length of chains, while red lines in processed images are region of interest scan lines at selected sections of images (b, e & h) which generated profile distributions shown in (c, f &i).

respectively, with an example shown in Table S-6 for all red lines (identified as scan profile). The estimated numbers were multiplied by 0.167 µm to obtain the dimensional thickness and gaps of chains. The average and standard deviation of the profiles are shown in Table S-7. The average values (Av) of the combined for scanned profile were reprocessed to obtain the ratio between thickness of chain and gap between them by Av/(1-Av).

Table-S-3: Example of scan plot profile for one of the red lines from Figure S7 with X as pixel divisions (167nm/pixel) and Y as the binary pixel intensity.

Red Line I		Red Line II		Red Line III		Red Line IV	
X (divisions-nm/pixel)	Y (pixel intensity)	X (divisions-nm/pixel)	Y (pixel intensity)	X (divisions-nm/pixel)	Y (pixel intensity)	X (divisions-nm/pixel)	Y (pixel intensity)
0.00E+00	0.00E+00	0.00E+00	0.00E+00	0.00E+00	2.55E+02	0.00E+00	0.00E+00
1.67E-01	0.00E+00	1.67E-01	0.00E+00	1.67E-01	2.55E+02	1.67E-01	2.55E+02
3.34E-01	0.00E+00	3.34E-01	2.54E+02	3.34E-01	2.55E+02	3.34E-01	2.55E+02
5.02E-01	5.71E+00	5.02E-01	2.53E+02	5.02E-01	2.55E+02	5.02E-01	0.00E+00
6.69E-01	2.55E+02	6.69E-01	2.53E+02	6.69E-01	2.53E+02	6.69E-01	2.55E+02

Table-S-4: Example of output from simple program (in Supplementary S1.11) that counted number of pixels in each chain block to identify chain thickness and chain gaps within the scanned profile. CntCH and CntGap is number of pixel counts for a chain and gap respectively.

Scan Profile I		Scan Profile II		Scan Profile III		Scan Profile IV	
Total No. Rows	805	Total No. Rows	857	Total No. Rows	931	Total No. Rows	885
CntCH	CntGap	CntCH	CntGap	CntCH	CntGap	CntCH	CntGap
4	3	3	2	6	5	2	1
5	2	4	14	1	5	9	1
4	11	3	2	2	5	2	3
8	6						

Table-S-5 Summary of results obtained from image analysis of sample prepared under 2500 rpm spin speed

**2500 RPM**									
**r (m)**	**CG (μm)**	**CT (μm)**	**CL (μm)**	**SD-CG**	**SD-CT**	**SD-CL**	**CL/CT**	**CT/CG**	**Qpi**
0.001	2.32	2.66	42.7	0.14	0.23	5.32	2320	1.15	13
0.0025	2.65	2.46	93	0.22	0.2	1.72	1060	0.93	5
0.0042	2.82	2.15	209	0.5	0.07	19.	672	0.73	2
0.0049	3.05	1.94	98	0.58	0.14	13.5	622	0.64	1.5
0.0057	5.03	1.215	1.21	0.32	0.076	0.34	883	0.24	1

SD represents Standard deviation

S1.15 Algorithm for Measuring the Chain thickness and gap Profile scan

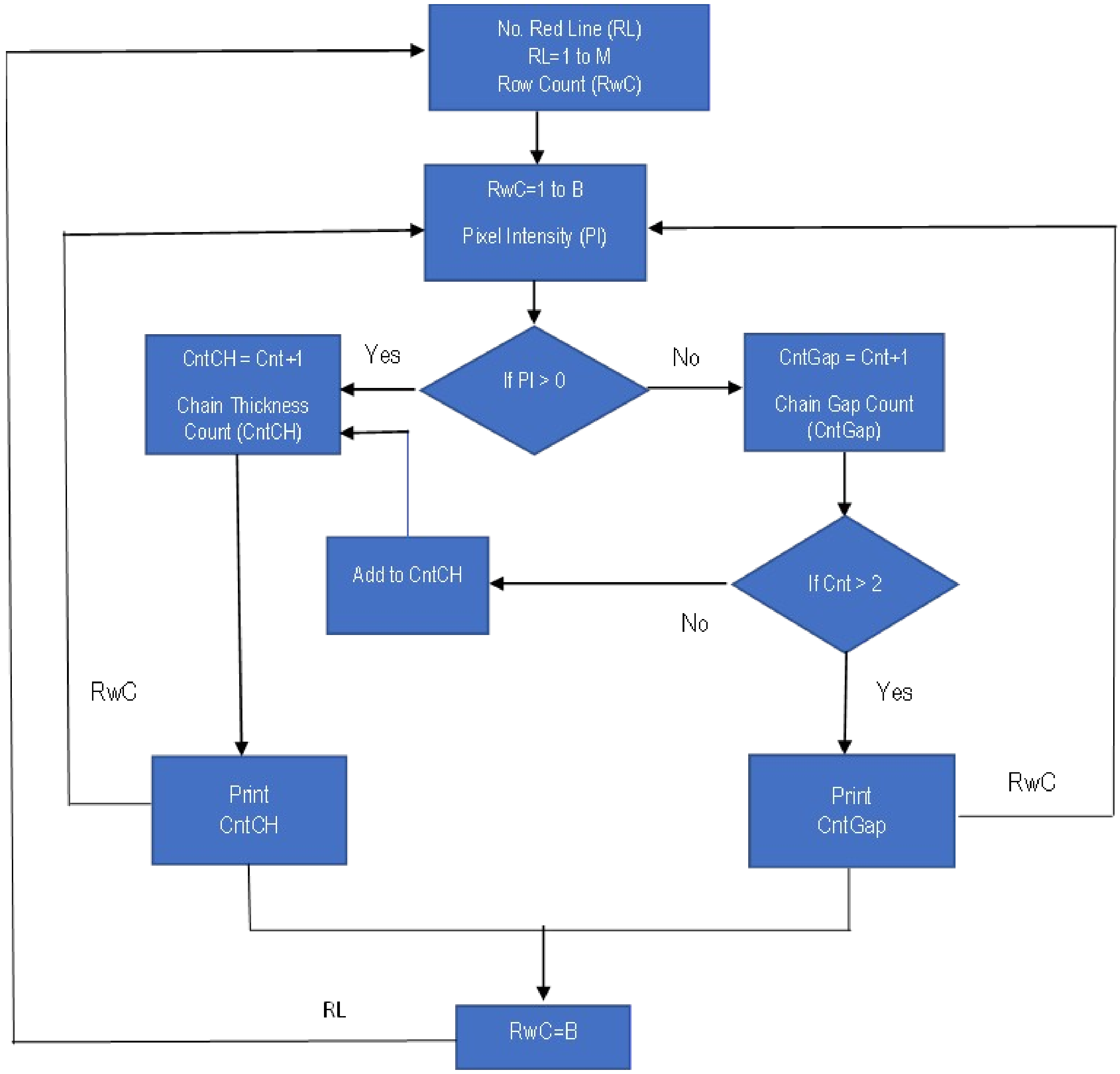

M is the Number of scan profile lines (red lines) which is 4 in this study, **B** is the number of total pixel points within each scan profile line. **RwC** is the count process across each pixrl point within the scan profile line.

S1.16 Specular Reflectance FTIR

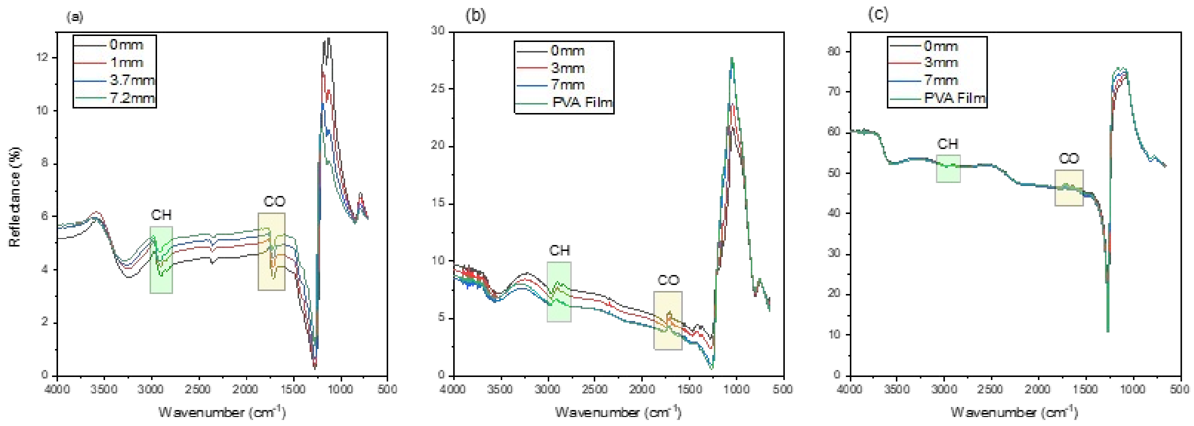

Figure S 8: Specular reflectance spectra of PEG-C-GM-FF in PVA thin film with range 4000 cm-1 to 1600 cm-1 at (a) 20°, (b) 45° and (c) 82°.

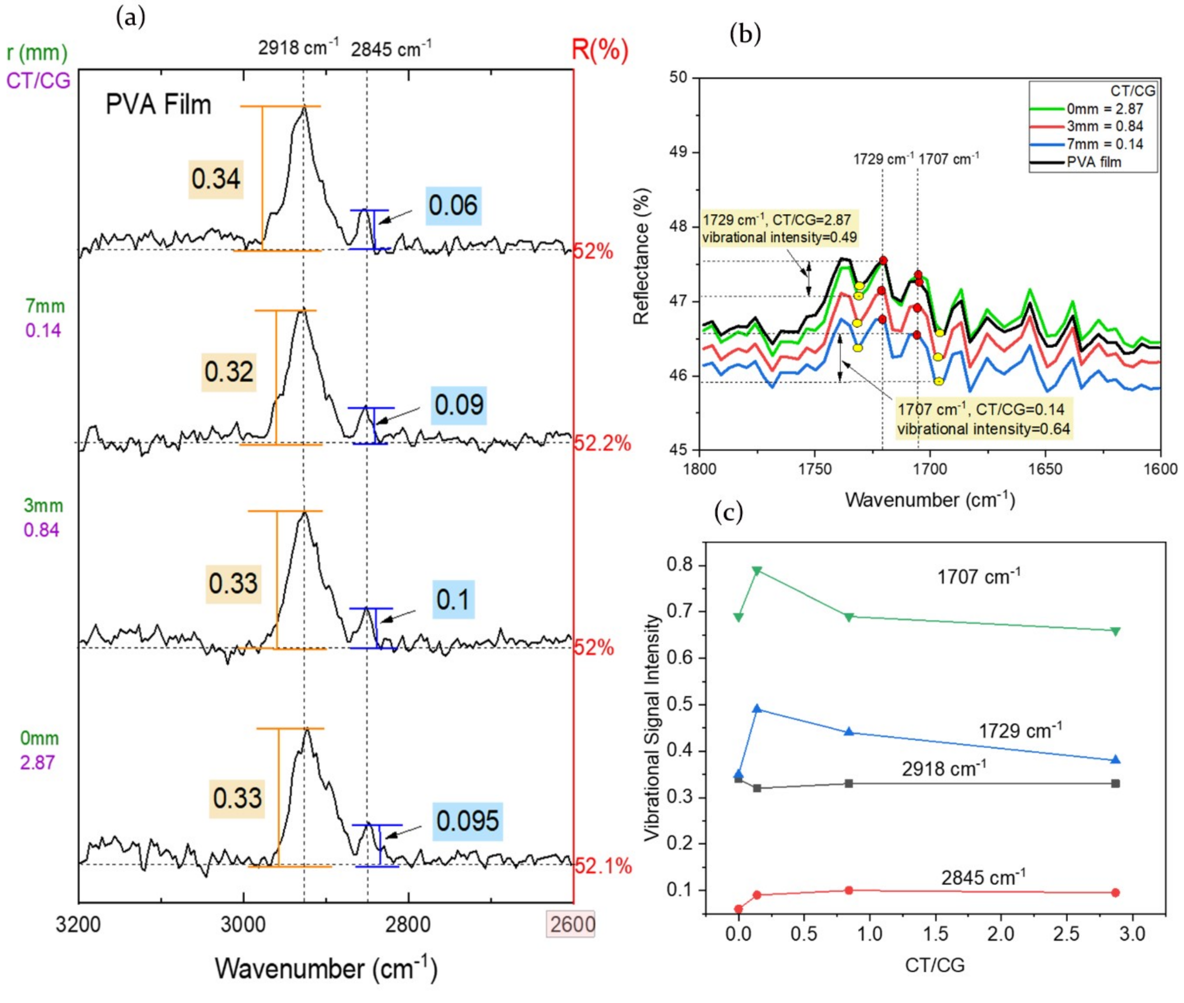

Figure S9: (a)Specular Reflectance spectra with range 3200 cm-1 to 2600 cm-1, identifying the change in vibration intensity of CH bonds – 2918cm^{-1} and 2845cm^{-1}, with 2600 cm^{-1} used as reference reflectance intensity point (R%) for 82°. (b) Specular Reflectance spectra with range 1900 cm^{-1} to 1600 cm^{-1}, identifying the change in vibrational intensity CO bonds, 1729 cm^{-1} and 1707 cm^{-1} for 82°, (c) vibrational signal for band 1707 cm^{-1}, 1729 cm^{-1}, 2845cm^{-1} and 2918cm^{-1} against change in CT/CG.

S1.17 Materials

Iron powder (fine), cis-Cyclooctene (Mw 110.2 g/mol), Hydrochloric acid (37% w/w molar concentration), Ammonium Hydroxide (30% wt. Ammonia in water), Hydrogen peroxide (30% w/w in water) Polyvinyl Alcohol (PVA) (Mw 88000 g/mol), Oleic acid (Mw 282.46 g/mol), Gold (III) chloride (30 wt. % in dilute HCl – Mw 339.79 g/mol), Sodium methacrylate (Mw 108.07 g/mol), Polyethylene Glycol 40 Stearate (PEG 40S). All chemical purchases were made from Sigma Aldrich. The resistivity of the deionised (DI) water used was 18 MΩ. Whatman glass microfibre filter paper (GF/A) and plain microscopic soda lime glass slides – 72mm X 24.5mm X 1mm (Agar Scientific) was used for filtration and substrate.

S1.17.1 Equipment

Thin film was prepared by using SCS™ 6800 spin coater. The external magnetic field was provided by square Neodymium Magnets (3 mm x 3mm x 3mm) purchased from First4magnets® UK. A gaussmeter model VTSYIQI® probe type having with measurement scope from 0～200mT - ～2000mT (1mT=10Gs); resolution 0.1mT (accuracy 1%), and operating temperatures -10°C～40°C was use to monitor magnetic field. 8100 Plus pH-temperature meter (ETI ltd), Perkin Elmer Lambda 850 UV-Vis absorption spectrophotometer, Eppendorf® centrifuge 5418, Olympus® BX41 Darkfield microscope, Quanta 650 FEG Scanning Electron Microscope, Thermo ScientificTMNicoletTMiSTM20 FTIR spectrometer, ThermoScientificTMNicoletTMiNTM 10 infra-red microscope, Agilent® 4100 ExoScan FTIR analyser, Malvern® Zetasizer Nano ZS instrument.

S1.18 Methods

S1.18.1 Oil based ferrofluid preparation

Initially, 20g of iron powder was dispersed in 100ml DI water followed by the addition of 100ml of 30 wt.% Hydrochloric acid to form green coloured iron (II) chloride solution. The solution was split into two equal parts and one part oxidized further into orange coloured iron (III) chloride solution by adding 5ml hydrogen peroxide. Both solutions were decanted using the glass microfibre filter paper to remove undissolved solids.2ml of Iron (II) chloride and 2.5ml of iron (III) chloride solutions were combined in a beaker containing 100ml of water. The solution was sonicated at 40kHz frequency in ultrasonic water bath maintained at 60oC while purged with nitrogen gas. Ammonium hydroxide was added dropwise into the solution until the pH rose from ~2 to ~9. Instrument 8100 Plus pH temperature meter (ETI Ltd) was use to monitor the pH. During the mixing process, the colour of the solution changed from light yellow to black confirming the (Fe3O4) magnetite precipitation. Further, 2.1g of oleic acid compound was mixed with 5ml of 30wt.% ammonium hydroxide to form ammonium oleate for the coating the magnetite NPs. The ammonium oleate solution was added to the prepared magnetite suspension and was stirred at 450 rpm with a rotor for 3.5 hours while the 65wt.% nitric acid was added dropwise to reduce the pH from ~9 to ~7 [4], [5], [6]. Afterwards, the magnetite NPs were precipitated and rinsed several times with isopropanol and deionized water to remove excess ammonia and ammonium salts, and then allowed to dry in vacuum oven at 40oC temperature. The process was repeated in batches to finally accumulate 5g of oleic acid

coated magnetite that was dispersed in 7ml of cis-Cyclooctene to prepare the oil based ferrofluid. The process is depicted in the 1st row of the Figure 1 in the main text of the article.

S1.18.2 Gold methacrylate colloid preparation

0.058mM of gold (III) chloride solution was added into a stirring 100ml DI water maintained at 90°C. In addition to this, the 12 ml of 2 wt.% aqueous solution of sodium methacrylate (0.407g in 20ml of DI water) was added at once into aqueous gold chloride solution, establishing a mole ratio between gold ions and methacrylate ions as 1:39. The solution turned from transparent to red wine colour after 15 minutes of stirring indicating the formation of gold methacrylate colloids. After the solution was cooled down to room temperature, 20µg/ml of polyethylene glycol (PEG 40S) was added to the solution and allowed to mix at 500rpm for 6 hours in a 200ml beaker sealed with aluminium foil. Finally, the solution was centrifuged at 11000rpm using an Eppendorf® centrifuge 5418 to separate the PEG coated gold methacrylate (PEG-C-GM) NPs pallets from the liquid. Obtained nanoparticle pallets were re-suspended in DI water and centrifuged at least four more times to remove sodium salts and excess PEG (non-bonded) molecules. The washed nanoparticles were redispersed in 15ml of DI water until further use. The concentration of gold NPs in DI water at this stage was 4.2±0.2 mg/L. The process is depicted in second row of the Figure 1 in the main text of the article.

S1.19 References

1) Thermo-Scientific, (2013). https://www.thermofisher.com/document-connect/document-connect.html?url=https://assets.thermofisher.com/TFS-Assets%2FCAD%2FSpecification-Sheets%2FNicolet%20iN10-PS51510.pdf
2) T. Kuroda, S. Chalimah, Y. Yao, N. Ikeda, Y. Sugimoto, K. Sakoda, Apparatus for high-precision angle-resolved reflection spectroscopy in the mid-infrared region. *Applied Spectroscopy*, *75*(3) (2021) 259-264.
3) M. Mohsen-Nia and H. Modarress, Viscometric study of aqueous poly(vinyl alcohol) PVA solutions as a binder in adhesive formulations. *J. Adhesion Sci. Technol.,* 20(12) (2006) 1273-1280.
4) H. Zhang, F. Kin-Hung, H. Jürgen, C. Che Ting and W. Dayang, Controlled chainlike agglomeration of charged gold nanoparticles via a deliberate interaction balance. *The journal of physical chemistry C*, *112*(43) (2008) 16830-16839.
5) H. Chen, L. Shao, Q. Li and J. Wang, Gold nanorods and their plasmonic properties. *Chemical Society Reviews*, *42*(7) (2013) 2679-2724.
6) W.L.F. Chin, T. Mun F. and H. Sharifa B. A., (2017), Adv. Polym. Technol., vol. 37, pp. 1712-1721.
